# PI3K/AKT/mTOR pathway-derived risk score exhibits correlation with immune infiltration in uveal melanoma patients

**DOI:** 10.3389/fonc.2023.1167930

**Published:** 2023-04-20

**Authors:** Yuxin Geng, Yulei Geng, Xiaoli Liu, Qiannan Chai, Xuejing Li, Taoran Ren, Qingli Shang

**Affiliations:** ^1^ Department of Ophthalmology, The Second Hospital of Hebei Medical University, Shijiazhuang, China; ^2^ Department of Ophthalmology, Shijiazhuang People’s Hospital, Shijiazhaung, China

**Keywords:** uveal melanoma, PI3K/Akt/mTOR pathway, prognostic-related risk model, machine learning, tumor immune infiltration

## Abstract

Uveal melanoma (UVM) is a rare but highly aggressive intraocular tumor with a poor prognosis and limited therapeutic options. Recent studies have implicated the PI3K/AKT/mTOR pathway in the pathogenesis and progression of UVM. Here, we aimed to explore the potential mechanism of PI3K/AKT/mTOR pathway-related genes (PRGs) in UVM and develop a novel prognostic-related risk model. Using unsupervised clustering on 14 PRGs profiles, we identified three distinct subtypes with varying immune characteristics. Subtype A demonstrated the worst overall survival and showed higher expression of human leukocyte antigen, immune checkpoints, and immune cell infiltration. Further enrichment analysis revealed that subtype A mainly functioned in inflammatory response, apoptosis, angiogenesis, and the PI3K/AKT/mTOR signaling pathway. Differential analysis between different subtypes identified 56 differentially expressed genes (DEGs), with the major enrichment pathway of these DEGs associated with PI3K/AKT/mTOR. Based on these DEGs, we developed a consensus machine learning-derived signature (RSF model) that exhibited the best power for predicting prognosis among 76 algorithm combinations. The novel signature demonstrated excellent robustness and predictive ability for the overall survival of patients. Moreover, we observed that patients classified by risk scores had distinguishable immune status and mutation. In conclusion, our study identified a consensus machine learning-derived signature as a potential biomarker for prognostic prediction in UVM patients. Our findings suggest that this signature is correlated with tumor immune infiltration and may serve as a valuable tool for personalized therapy in the clinical setting.

## Introduction

Uveal melanoma (UVM) is a rare yet malignant tumor that primarily affects the eyes of adults and poses a significant risk to their visual function and survival (1). This tumor arises from uveal melanocytes and is mainly observed in the choroid, typically in a unilateral manner (2). The incidence of UVM varies geographically and racially, with white populations exhibiting the highest incidence, followed by yellow populations, and black populations exhibiting the lowest incidence (3). Notably, around half of all UVM patients develop hematogenous metastasis, with 90% of these metastases occurring in the liver (4). Despite recent advances in treatment strategies, the overall survival rate of UVM patients has remained poor, with a median survival of 6-12 months after metastasis (5). Thus, there is an urgent need to identify novel prognostic biomarkers and predictive models for UVM patients.

The PI3K/AKT/mTOR signaling pathway plays a crucial role in regulating various cellular processes, including proliferation, transcription, translation, apoptosis, and angiogenesis, and is associated with mammalian tumor immunity (6–8). Dysregulated activation of this pathway is commonly observed in tumorigenesis and promotes drug resistance and tumor cell survival (9, 10). However, the potential significance of PI3K/AKT/mTOR pathway-related genes (PRGs) in the context of UVM remains to be fully elucidated. The PI3K/AKT/mTOR signaling pathway has emerged as a critical player in the regulation of tumor immune microenvironment (TME). This pathway plays a pivotal role in the modulation of a variety of immune cells, including T cells, B cells, and natural killer cells, as well as in the production of cytokines and chemokines (11–14). Moreover, dysregulation of this pathway has been implicated in the development of immune escape mechanisms in tumors, which in turn, can facilitate tumor growth and progression. Understanding the complex interplay between the PI3K/AKT/mTOR pathway and the tumor immune microenvironment will undoubtedly provide critical insights for the development of novel immunotherapeutic strategies for UVM treatment.

In this study, we conducted unsupervised clustering on 14 PRGs profiles to classify UVM samples into three clusters with distinct immune characteristics. Finally, we identified a consensus PRGs signature using 76 algorithm combinations through systematic machine learning analysis. Importantly, specific subtypes is correlated with tumor immune infiltration and activation of PI3K/AKT/mTOR signaling pathway. In the future, our results may serve as a valuable tool for personalized therapy in the clinical setting.

## Materials and methods

### Data acquisition

Uveal melanoma (UVM) mRNA gene expression profiles were acquired from The Cancer Genome Atlas (TCGA) and Gene Expression Omnibus (GEO) databases. The inclusion criteria consisted of complete follow-up information, non-zero survival days, and non-repeated sequencing. The meta-cohort analysis included 80 tumor samples from the TCGA-UVM cohort and 63 tumor samples from the GSE22138 cohort. Somatic mutation data from the TCGA-UVM cohort was incorporated. The batch effect between RNA-seq and microarray data was removed using the “sva” package to create a meta-cohort. A total of 105 pathway-related genes (PRGs) were obtained from the HALLMARK PI3K AKT MTOR SIGNALING entry in the Gene Set Enrichment Analysis (GSEA) database and previous literature (15).

### Clustering analysis

In order to evaluate the predictive capability of PRGs, a univariate Cox regression analysis was conducted within the meta-cohort. The expression levels of these PRGs were then utilized to establish the optimal number of clusters *via* unsupervised consensus clustering analysis, utilizing the “consensusClusterPlus” package. The efficacy of this clustering method was subsequently evaluated using principal component analysis (PCA). To determine statistical significance, the Kaplan-Meier curve was employed to assess overall survival (OS) in Uveal Melanoma (UVM) patients within the dataset, with the log-rank test being applied.

### Functional analysis

To discern Differentially Expressed Genes (DEGs) between UVM subtypes, the “limma” package was employed, with genes exhibiting an absolute log-fold change greater than 1 and a p-value less than 0.05 being deemed significant. Further annotation of these DEGs was achieved *via* the utilization of the Kyoto Encyclopedia of Genes and Genomes (KEGG) pathway analysis with the “clusterProfiler” package, with a threshold of p-value and false discovery rate (FDR) q-value less than 0.05 to identify pathways exhibiting significant enrichment. To evaluate biological pathway disparities between subtypes, gene set variation analysis (GSVA) was performed using the h.all.v7.4.symbols gene set, with a FDR threshold of less than 0.05.

### Potential therapeutic agent prediction

The calculation of IC50 was conducted using the “prophetic” package within the R software environment. Relevant drugs, including small molecule drugs for PI3K/KT/mTOR (A.443654, AKT.inhibitor.VIII, JW.7.52.1, MK.2206), were acquired from the Genome of Drug Sensitivity in Cancer (GDSC) database. The “pRRophetic” package leverages cell line expression profiles from large-scale projects, along with corresponding IC50 data, as primary inputs. Employing ridge regression, a predictive model is constructed and subsequently applied for the prediction of Chemotherapeutic Response in clinical samples.

### Immune microenvironment analysis

In order to gauge immune cell abundance in the samples, multiple algorithms were employed. The ESTIMATE algorithm was utilized to determine the immune score and stromal score, which were employed as indicative metrics of the overall immune microenvironmental status. Moreover, Pearson correlation analysis was performed to evaluate the relationship between key PRGs and immune score.

### Signature generated from machine learning integrative approaches

The current study builds on a previous workflow (16), whereby a combination of 76 machine learning algorithms were developed using the Lasso, RSF, GBM, Survival-SVM, SuperPC, ridge, plsRcox, StepCox, and Enet models. A pre-model that performed variable filtering was selected, and the prognostic PRGs expression profile file from the TCGA-UVM cohort was utilized to create a signature. The risk score was subsequently calculated in both the GSE22138 and full cohorts, and the best prognostic model was chosen based on the average C-index of the cohort. Finally, the predictive accuracy of the risk score was assessed *via* the plotting of a ROC curve.

### Cell culture

The RGC-5 and D407 cell lines were procured from ATCC and LY294002 (Cat no. L9908) was sourced from Sigma Inc. Both cell lines were cultured in DMEM (Gibco) containing 10% fetal bovine serum (Gibco), at 37°C and 5% CO2. These cell lines are extensively utilized in ocular disease research, such as glaucoma and age-related macular degeneration, due to their shared molecular and phenotypic characteristics with UVM cells (17–19). In line with previous studies, we selected LY294002 concentrations of 0μM, 5μM and 10μM for the experiments (20). Cell proliferation was assessed by treating the cells with LY294002 for 24 hours and then conducting a Cell Counting Kit-8 (CCK-8) assay (Dojindo, Kumamoto, Japan) to measure cell viability.

### Statistical analysis

The statistical analyses were executed using R software (version 4.0.1), as described earlier in this study. The significance level was set at a p-value below 0.05, indicating statistical significance.

## Results

### Expression of PRGs in UVM and univariate cox analysis

**Figure 1:**
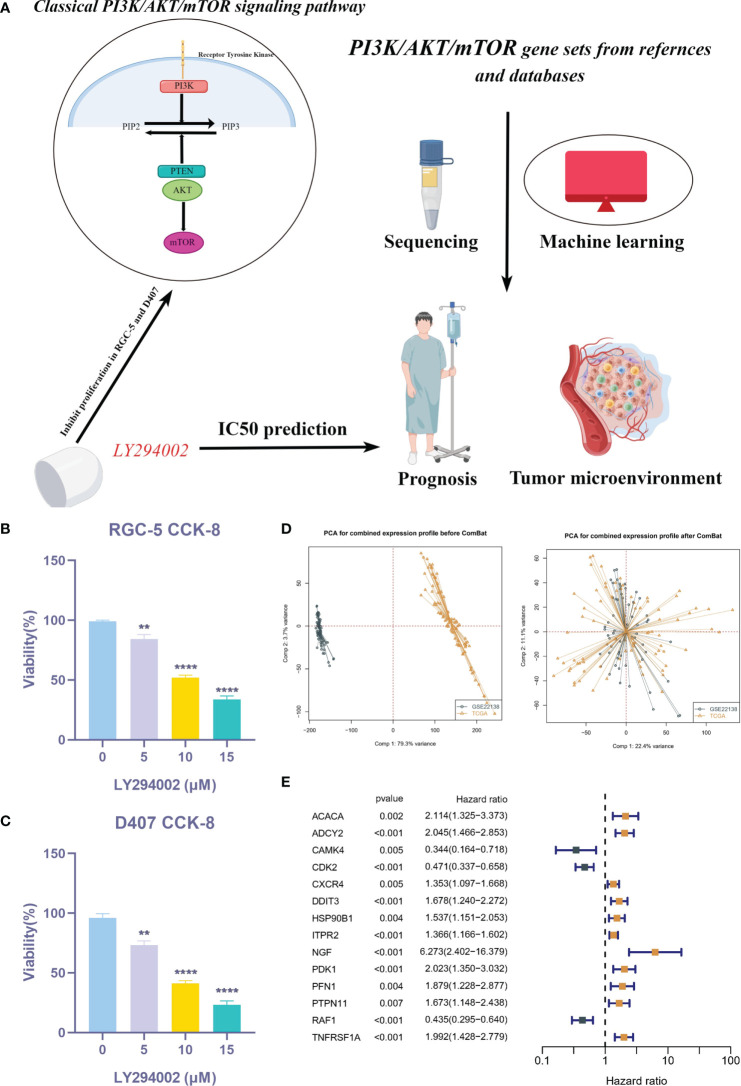
Expression of PRGs in UVM and univariate Cox analysis. **(A)** Flow chart of this study. **(B, C)** CCK-8 assay. **(D)** PCA analysis in TCGA cohort and GSE22138 cohort. **(E)** The univariate Cox analysis of PRGs in UVM. **P < 0.01, ****P < 0.0001.

**Figure 1A** illustrates the flow chart of this study. In order to clarify the effect of PI3K/AKT/mTOR pathway on UVM cells, we used CCK-8 kit to detect cell viability in RGC-5 and D407 cells. **Figures 1B, C** showed that the cell viability of LY294002 treatment group was lower than that of control group, and the cell viability decreased with the increase of LY294002 concentration. To further explore the critical role of PRGs in UVM patients, we used the combat algorithm to remove batch effects from different cohort expression profiles (**Figure 1D**). In the meta cohort, 14 genes were associated with prognosis (**Figure 1E**).

### Molecular subtype identification

**Figure 2:**
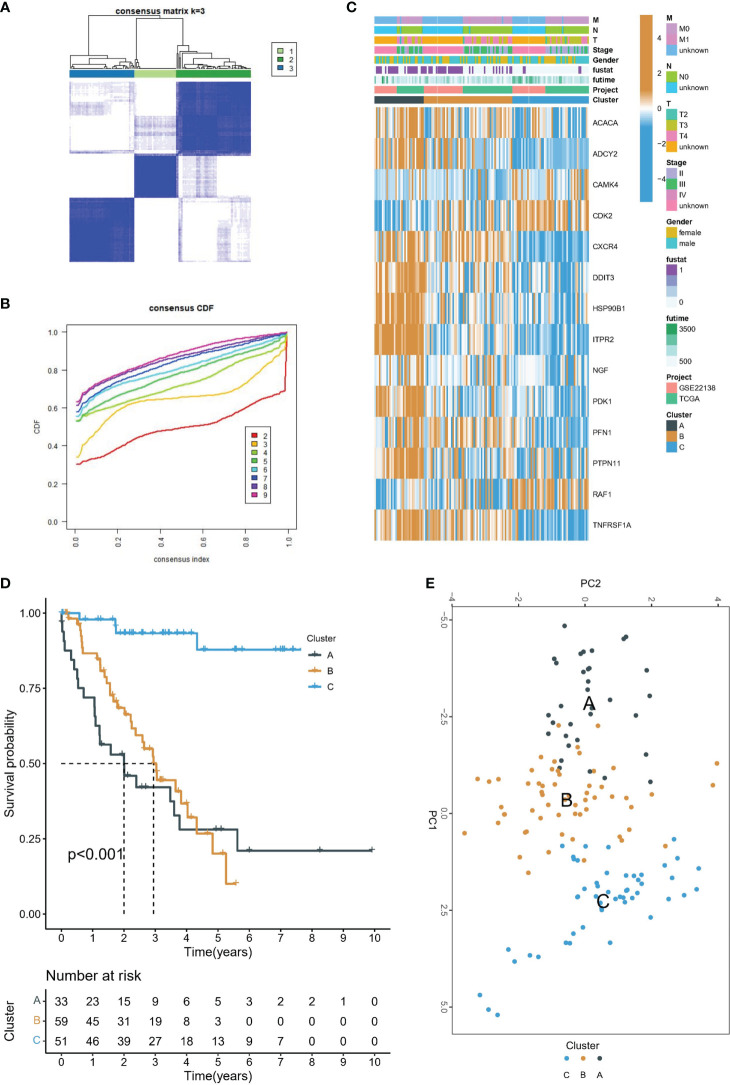
Molecular subtype identification. **(A)** Consensus clustering matrix when k = 3. **(B)** Consensus clustering CDF with k valued 2 to 9. **(C)** The heat map shows the correlation between the two clusters and the clinicopathological features. **(D)** Kaplan-Meier curves of OS for three subtypes in UVM. **(E)** PCA analysis showed that UVM could be well differentiated into three subtypes based on the expression of 14 prognostic PRGs.

In the meta-cohort study, patients were categorized according to the consistent clustering of 14 prognostic PRGs. Notably, a cluster selection of 3 yielded a relatively stable clustering result, as demonstrated in **Figures 2A, B**. Finally, we obtained three molecular subtypes. A heat map of clinical information and gene expression was constructed, and we found that 14 prognostic PRGs were significantly up-regulated in subtype A (**Figure 2C**). There were significant prognostic differences among the three molecular subtypes, with subtype A having the worst prognosis and subtype C having the best prognosis (**Figure 2D**). Finally, PCA showed genomic heterogeneity in different cohorts (**Figure 2E**).

### Relationship between PI3K/AKT/mTOR clusters and tumor microenvironment and drug sensitivity

**Figure 3:**
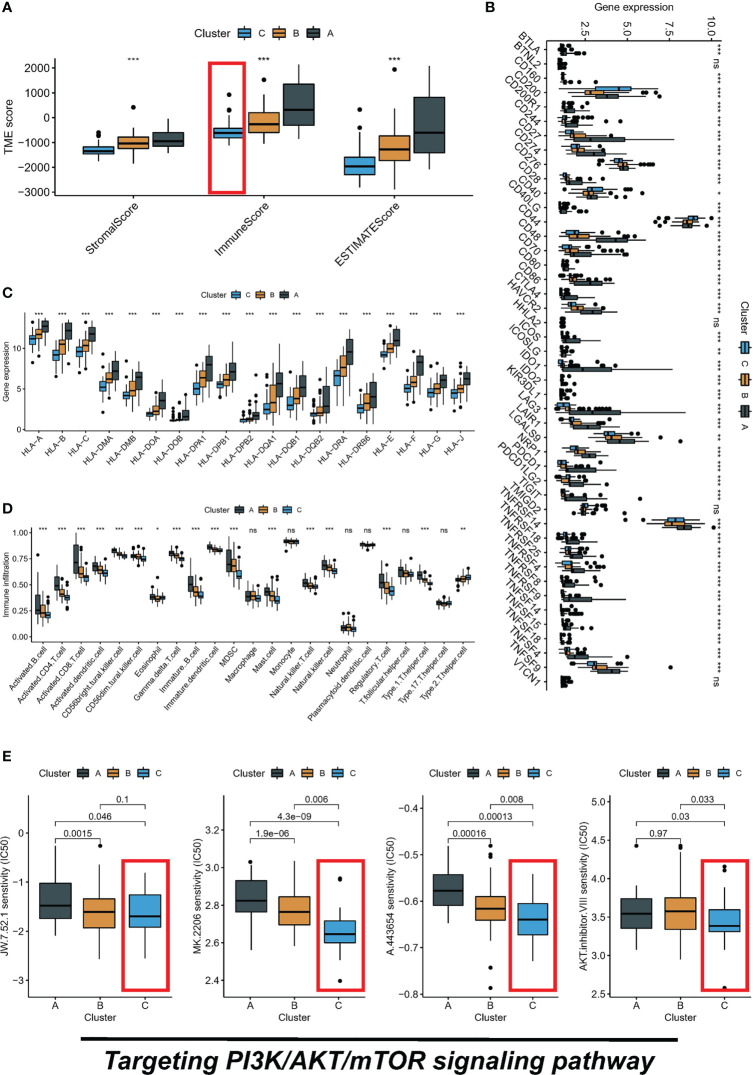
Three subtypes display different immune landscape and chemotherapy drug sensitivity. **(A)** The expression level of the immune score, stromal score, and ESTIMATE score, between different subtypes. **(B)** Comparison of immune checkpoint blockade-related genes expression levels in three subtypes. **(C)** Comparison of 19 HLA-related genes expression levels in three subtypes. **(D)** immune function by ssGSEA. **(E)** The boxplot of sensitivity of small molecule drugs targeting PI3K/AKT/mTOR between three subtypes. ns, not significant, *P < 0.05, **P < 0.01, ***P < 0.001.

The PI3K/AKT/mTOR signaling pathway has emerged as a critical player in the regulation of tumor immune microenvironment. The ESTIMATE algorithm evaluated the overall TME landscape for different subtypes, with subtype C having the lowest immune and stromal scores and subtype A having the highest immune and stromal scores (**Figure 3A**). Furthermore, we conducted a comparative analysis of HLA molecule and ICI mRNA expression across different molecular subtypes and noted that subtype A exhibited elevated levels of both HLA and ICI mRNA, as depicted in **Figures 3B, C**. Additionally, TME status was assessed in the various molecular subtypes through ssGSEA analysis. Despite subtype A displaying the highest immune score according to the ESTIMATE algorithm, a significantly greater abundance of Treg cells was observed in subtype A compared to the other subtypes. This finding means that subtype A may appear to be in a “hot tumor” state, but it is in an immunosuppressed state, which may also be the main reason for poor prognosis (**Figure 3D**). Finally, we calculated the IC50 of small molecule drugs targeting PI3K/AKT/mTOR: A.443654, AKT.inhibitor.VIII, JW.7.52.1, MK.2206 from the GDSC database. The results showed that subtype C was the most sensitive to the mentioned drugs (**Figure 3E**).

### Biological features of molecular subtypes

**Figure 4:**
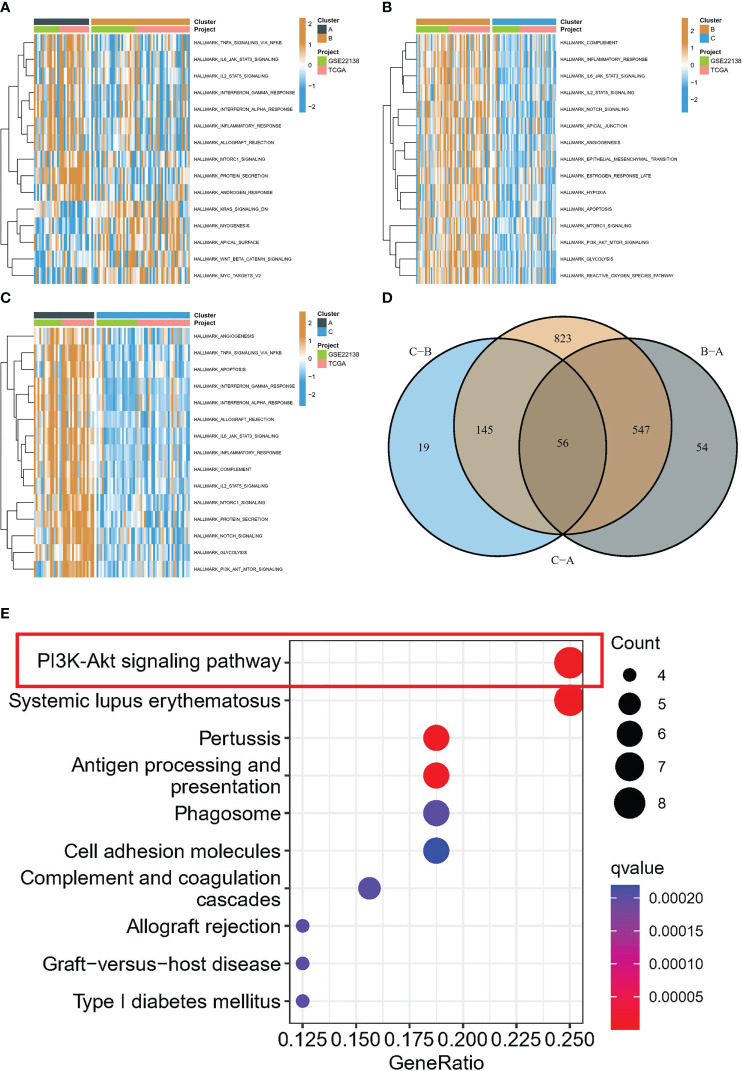
Biological characterization of molecular subtypes. **(A-C)** The GSVA pathway enrichment analysis between different subtypes. **(D)** Venn diagram of molecular subtype-associated DEGs. **(E)** KEGG analysis.

The association between the PI3K/AKT pathway and apoptosis and angiogenesis is widely recognized. In order to investigate this relationship, we conducted GSVA enrichment analysis using the Hallmark gene set. This analysis revealed that subtype B was significantly enriched in inflammatory response, apoptosis, angiogenesis, and the PI3K/AKT/mTOR signaling pathway compared to subtype C, as illustrated in **Figure 4B**. Subtype A demonstrated significant enrichment in inflammatory response, apoptosis, angiogenesis, and the PI3K/AKT/mTOR signaling pathway compared to subtype C, as displayed in **Figure 4C**. Similarly, subtype A was also significantly enriched in these processes when compared to subtype B, as depicted in **Figure 4A**. Furthermore, differential analysis was conducted between the different subtypes, resulting in the identification of 56 DEGs (**Figure 4D**). Intriguingly, KEGG analysis further revealed that the major enrichment pathway of these 56 DEGs was associated with the PI3K/AKT/mTOR signaling pathway (**Figure 4E**).

### Integrative construction of a consensus signature

**Figure 5:**
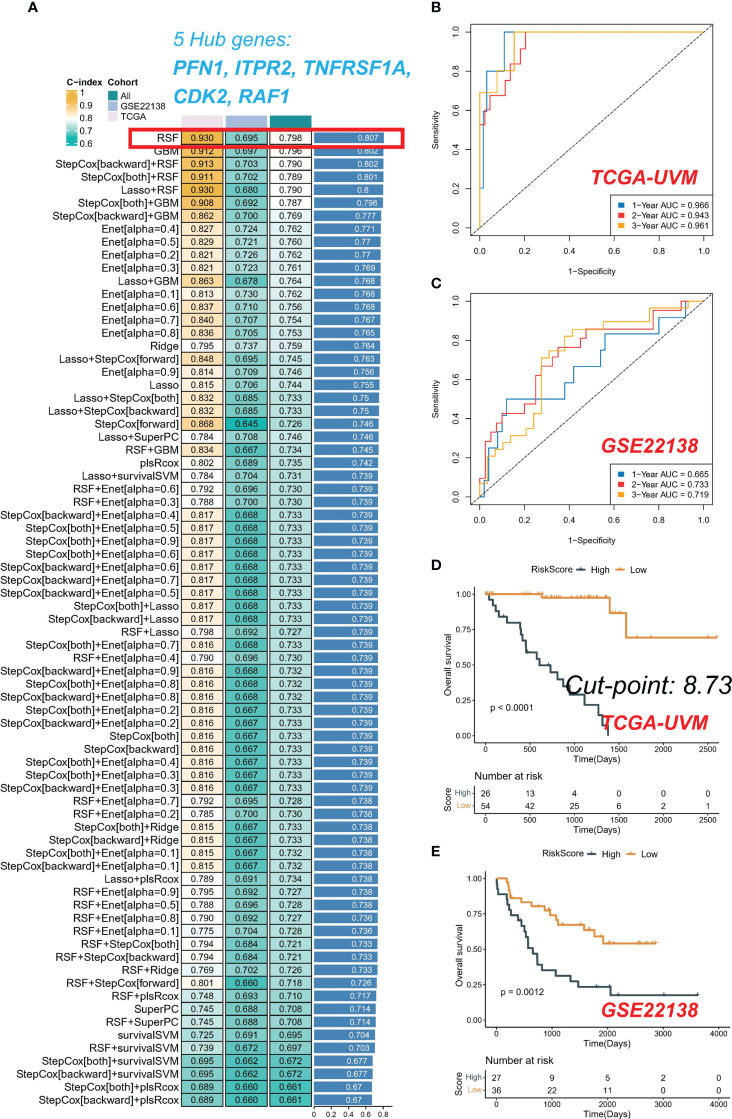
Integrative construction of a consensus signature. **(A)** C-indices of 76 kinds of prediction models in three cohorts. ROC analysis at 1, 2, and 3 years in TCGA-UVM **(B)** and GSE22138 **(C)**. Kaplan-Meier curves of OS in TCGA-UVM **(D)** and GSE22138 **(E)**.

The average C-index for each algorithm in all cohorts was calculated, and the final model was selected as the RSF algorithm with the highest average C-index (0.807), as depicted in **Figure 5A**. The risk score of each sample in the cohort was further calculated based on the expression files of the five PRGs (PFN1, ITPR2, TNFRSF1A, CDK2, and RAF1) contained in the RSF model. Subsequently, patients in the TCGA-UVM and GSE22138 cohorts were grouped using the optimal critical value of 8.73 determined based on the risk score of the training set. The GSE22138 cohort consisted of 27 high-risk and 36 low-risk patients, while the TCGA-UVM cohort comprised 26 high-risk and 54 low-risk patients. It is noteworthy that the training cohort demonstrated an excellent performance with an area under the ROC curve (AUC) of 0.966, 0.943, and 0.961 for 1-year, 2-year, and 3-year OS, respectively (**Figure 5B**). In the validation cohort, the AUC for 1-year, 2-year, and 3-year OS was 0.665, 0.733, and 0.719, respectively (**Figure 5C**). Kaplan-Meier curves indicated that patients in the low-risk group had significantly prolonged survival in both the training and test cohorts (**Figures 5D, E**).

### Evaluation of TME in high- and low-risk groups

**Figure 6:**
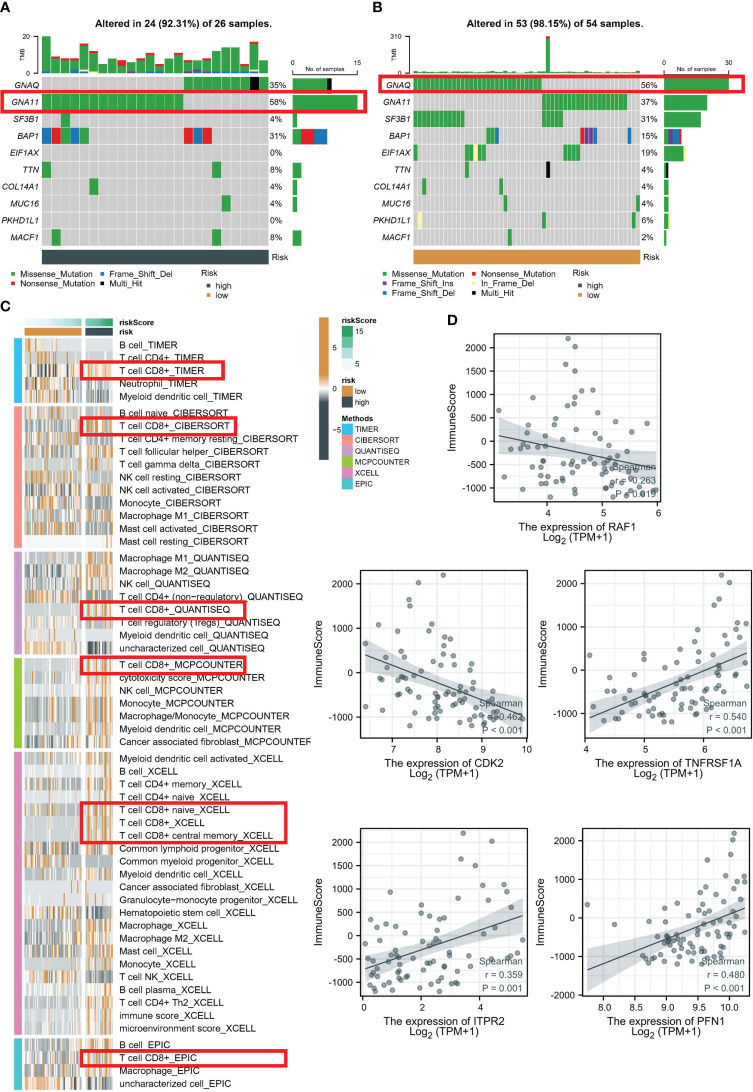
Evaluation of TME in high- and low-risk groups. Top 10 gene mutations in high- **(A)** and low-risk **(B)** groups. **(C)** Analysis based on different algorithms showed immune cell infiltration in low-risk and high-risk groups. **(D)** Pearson correlation analysis between five PRGs and immune score.

Upon analyzing the whole exome sequencing data, we found that there was a significant difference in the top mutation genes between the high-risk and low-risk groups. In the high-risk group, GNA11 was identified as the top 1 mutation gene (**Figure 6A**), while in the low-risk group, GNAQ was identified as the top 1 mutation gene (**Figure 6B**). Moreover, to estimate the infiltration fraction of immune cells in different samples, we performed immune cell analysis using various algorithms such as TIMER, CIBERSORT, QUANTISEQ, MCP-counter, XCELL, and EPIC. The results showed that the high-risk group had a more active tumor microenvironment (TME) (**Figure 6C**), with all algorithms indicating higher levels of immune killer cell CD8+ T in the high-risk group. Finally, to further explore the significance of the five PRGs involved in the RSF model and immunization, we conducted Pearson correlation analysis. Our findings demonstrated that RAF1 and CDK2 were significantly negatively correlated with immune score, whereas PFN1, ITPR2, and TNFRSF1A were significantly positively correlated with immune score (**Figure 6D**).

## Discussion

With the advent of high-throughput sequencing technology, a multitude of tumor genomics investigations have corroborated the aberrant activation of the PI3K/AKT/mTOR signaling pathway in malignant tumors (21–23). The activation state of various effector molecules is regulated by this pathway, which not only influences cell proliferation and apoptosis but also contributes to tumor growth and chemotherapy response (24, 25). Research has demonstrated that the PI3K/AKT/mTOR signaling pathway is linked to drug resistance in certain tumors, such as leukemia, prostate cancer, colon cancer, and lymphoma. Meanwhile, the use of inhibitors and traditional chemotherapy drugs targeting this pathway has exhibited enhanced chemotherapy efficacy and decreased drug resistance in the chemotherapy of arsenic tumors, such as leukemia (26, 27). However, the specific mechanism of PRGs in the onset, progression, and prognosis of UVM necessitates further exploration.

We have delineated three distinct subtypes on the basis of prognosis-related PRGs. These subtypes exhibit significant variations in terms of prognostic features, TME, and functional enrichment. The patients belonging to subtype A manifest the poorest prognosis and harbor more advanced clinicopathological features. To assess the overall TME landscape of the various molecular subtypes, we employed the ESTIMATE algorithm in our study. We noted remarkable variations in TME characteristics, such as stromal score, immune score, and ESTIMATE score, with subtype A demonstrating the highest immune and stromal scores. Additionally, we scrutinized the expression levels of HLA molecules and ICI mRNA across the diverse subtypes and detected higher levels of HLA and ICI mRNA expression in subtype A. Moreover, subtype A displayed the highest abundance of infiltrating immune cells among the different subtypes.

Notably, the PI3K/AKT/mTOR signaling pathway exhibited significant enrichment in subtype A, which is recognized for its involvement in the regulation of the tumor immune microenvironment. In particular, it has been demonstrated to modulate the secretion of immunosuppressive cytokines, expression of PD-L1, and infiltration of regulatory T cells and CD8+ T cells (13, 28). Furthermore, KEGG pathway analysis of 56 differentially expressed genes (DEGs) revealed that the principal pathways of enrichment were associated with the PI3K/AKT/mTOR signaling pathway. These findings suggest that targeting this pathway presents a promising approach to manipulate the tumor immune microenvironment and ameliorate clinical outcomes in patients with UVM.

We computed the mean C-index for each algorithm across all cohorts and selected the RSF algorithm as the final model due to its highest mean C-index (0.807). We subsequently determined the risk score of each sample in the cohort, based on the expression data of the five PRGs included in the RSF model: PFN1, ITPR2, TNFRSF1A, CDK2, and RAF1. Using the training set, we established the optimal cutoff value of 8.73 based on the risk scores, and implemented this cutoff to group patients in the TCGA-UVM and GSE22138 cohorts. Notably, the GSE22138 and TCGA-UVM cohorts comprised 27 high-risk and 36 low-risk patients, and 26 high-risk and 54 low-risk patients, respectively. In the training cohort, it is noteworthy that the AUC for 1-year, 2-year, and 3-year OS was 0.966, 0.943, and 0.961, respectively. In the validation cohort, the AUC for 1-year, 2-year, and 3-year OS were 0.665, 0.733, and 0.719, respectively. The Kaplan-Meier analysis indicated that the low-risk group had significantly longer survival compared to the high-risk group in both the training and validation cohorts. The current investigation delved deeper into the biological mechanisms that underlie the RSF model. The patients classified as high-risk exhibited a heightened activation of the TME, which may account for their increased rates of recurrence and mortality. In contrast, low-risk patients were distinguished by immune activation, which corresponded to more favorable prognostic outcomes. Moreover, the increased immune infiltrations observed in low-risk patients suggest that they may be well-suited candidates for immunotherapy.

Despite the promising findings of this study, there are several limitations that must be acknowledged. Firstly, RGC-5 and D407 are commonly used cell lines in research related to retinal diseases. RGC-5 is a rat retinal ganglion cell line, while D407 is a human retinal pigment epithelial cell line. These cell lines have been shown to share some molecular and phenotypic characteristics with UVM cells and have been used in several previous studies. However, we understand the limitations of using cell lines that do not perfectly reflect the biology of UVM and the need for more physiologically relevant models for preclinical research. In the future, we will aim to incorporate additional models, such as primary UVM cells and patient-derived xenografts, to further validate our findings and improve the relevance of our research to UVM. Secondly, the sample size of this study is relatively small, and larger studies are needed to validate the findings and improve the accuracy of the machine learning model.

## Conclusions

In summary, this study identified differentially expressed PRGs in UVM and classified UVM patients into three clusters based on the expression of prognostic PRGs. These clusters showed significant differences in prognosis and TME. A consensus PRG signature, termed RSF model, was systematically identified that could independently predict the prognosis and TME of UVM patients. The RSF model may serve as a robust predictor of prognosis and response to immunotherapy, providing a foundation for further personalized therapeutic strategies in UVM patients.

## Data availability statement

The original contributions presented in the study are included in the article/**Supplementary Material**. Further inquiries can be directed to the corresponding author.

## Ethics statement

Ethical review and approval were waived for this study, due to the use of open-accessed data.

## Author contributions

YXG and QS conceived and designed the study. YLG, XLL, and QS carried out experiments and gathered data. QC, XJL, and TR carried out data analysis and statistical analysis. The manuscript was written by YXG and YLG. All authors contributed to the article and approved the submitted version.
