# Supplementary figures and images for: PI3K/AKT/mTOR pathway-derived risk score exhibits correlation with immune infiltration in uveal melanoma patients
Source: Front Oncol. 2023 Apr 20;13:1167930. doi: 10.3389/fonc.2023.1167930 (PMC10157141; doi:10.3389/fonc.2023.1167930)

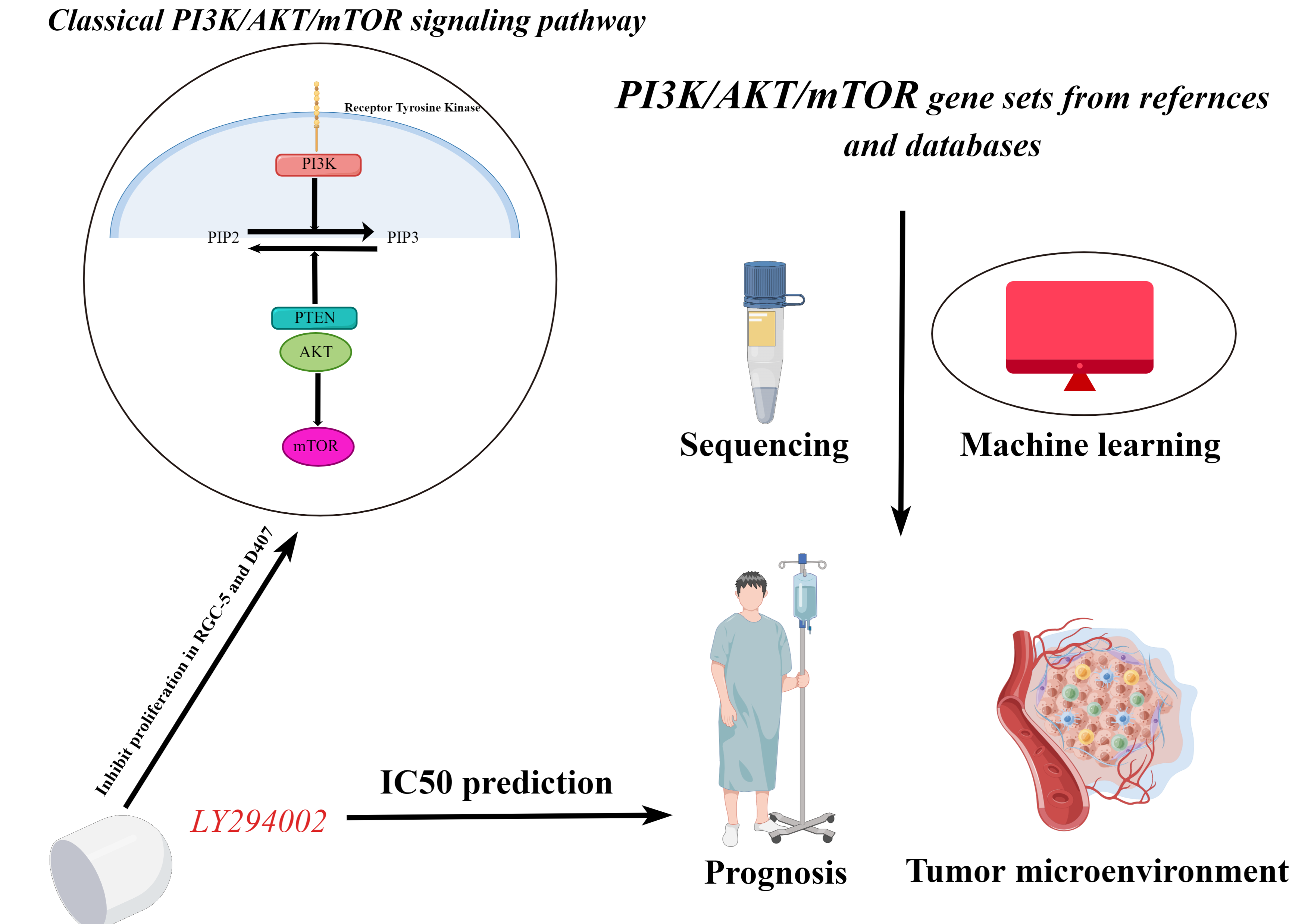

Supplement: Supplementary file 1 [file DataSheet_1.zip › raw data and Rcode for checking/export (1).png]

|          | pvalue | Hazard ratio        |
|----------|--------|---------------------|
| ACACA    | 0.002  | 2.114(1.325–3.373)  |
| ADCY2    | <0.001 | 2.045(1.466–2.853)  |
| CAMK4    | 0.005  | 0.344(0.164–0.718)  |
| CDK2     | <0.001 | 0.471(0.337–0.658)  |
| CXCR4    | 0.005  | 1.353(1.097–1.668)  |
| DDIT3    | <0.001 | 1.678(1.240–2.272)  |
| HSP90B1  | 0.004  | 1.537(1.151–2.053)  |
| ITPR2    | <0.001 | 1.366(1.166–1.602)  |
| NGF      | <0.001 | 6.273(2.402–16.379) |
| PDK1     | <0.001 | 2.023(1.350–3.032)  |
| PFN1     | 0.004  | 1.879(1.228–2.877)  |
| PTPN11   | 0.007  | 1.673(1.148–2.438)  |
| RAF1     | <0.001 | 0.435(0.295–0.640)  |
| TNFRSF1A | <0.001 | 1.992(1.428–2.779)  |

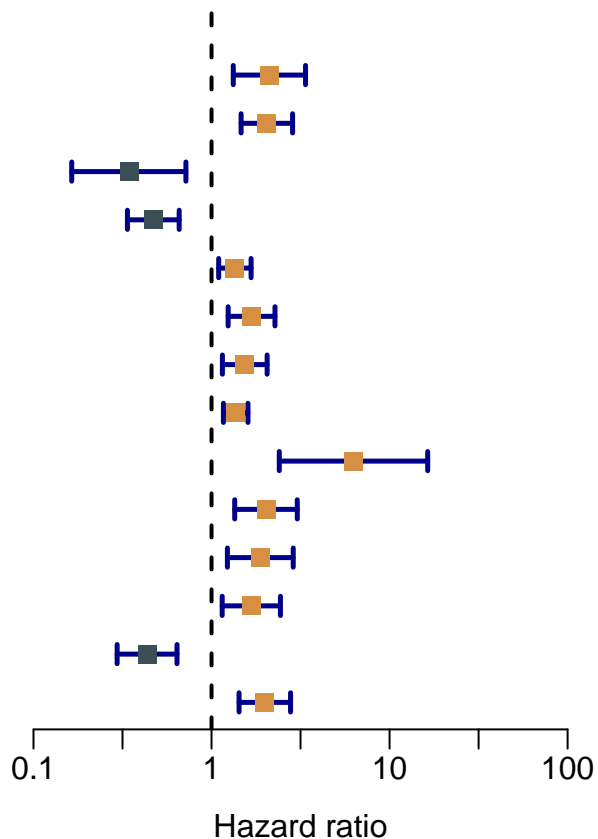

Supplement: Supplementary file 1 [file DataSheet_1.zip › raw data and Rcode for checking/FIg1/forest.pdf]

# PCA for combined expression profile after ComBat

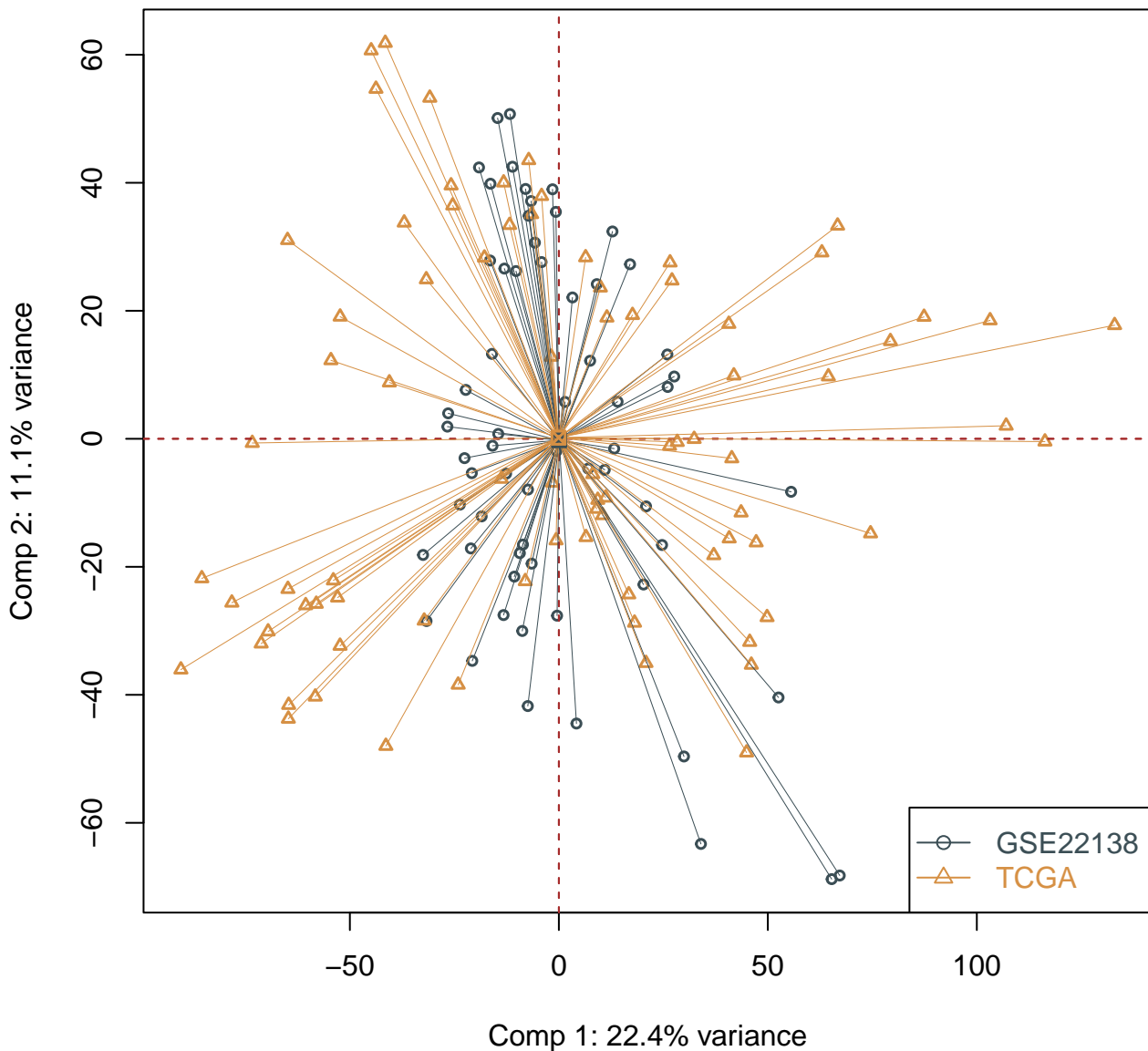

Supplement: Supplementary file 1 [file DataSheet_1.zip › raw data and Rcode for checking/FIg1/PCA for combined expression profile after ComBat.pdf]

# PCA for combined expression profile before ComBat

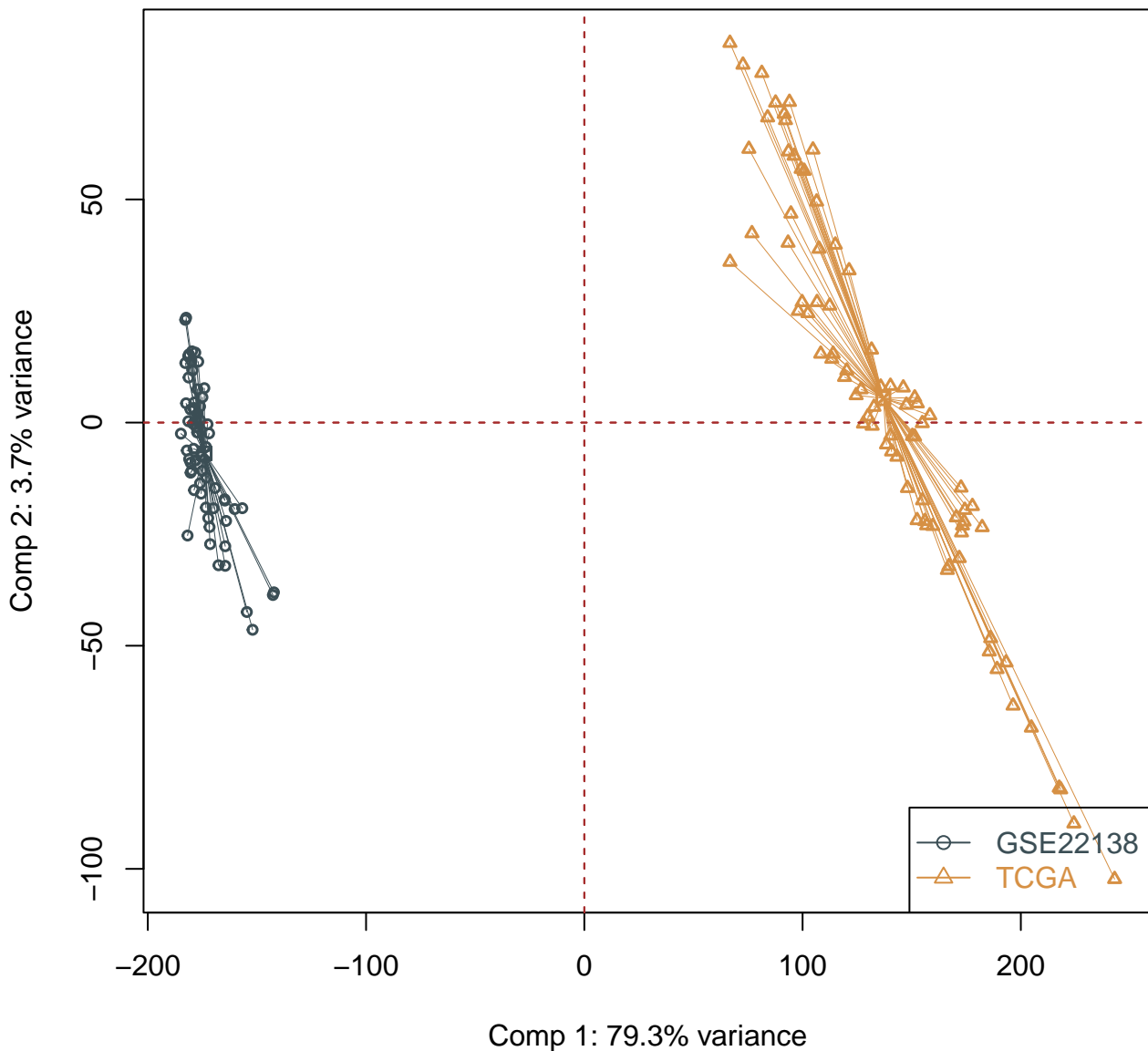

Supplement: Supplementary file 1 [file DataSheet_1.zip › raw data and Rcode for checking/FIg1/PCA for combined expression profile before ComBat.pdf]

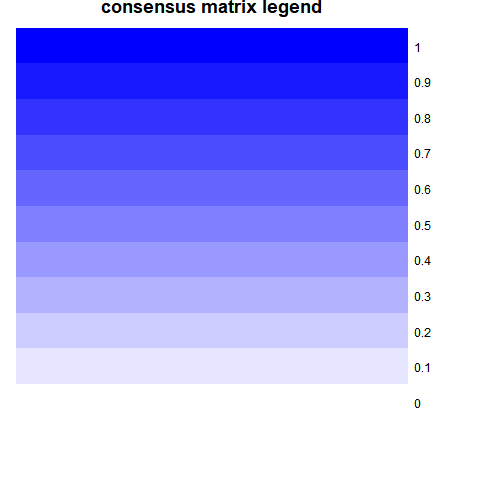

Supplement: Supplementary file 1 [file DataSheet_1.zip › raw data and Rcode for checking/Fig2/consensus001.png]

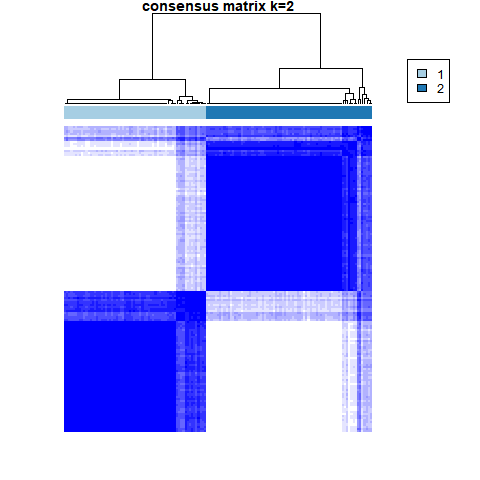

Supplement: Supplementary file 1 [file DataSheet_1.zip › raw data and Rcode for checking/Fig2/consensus002.png]

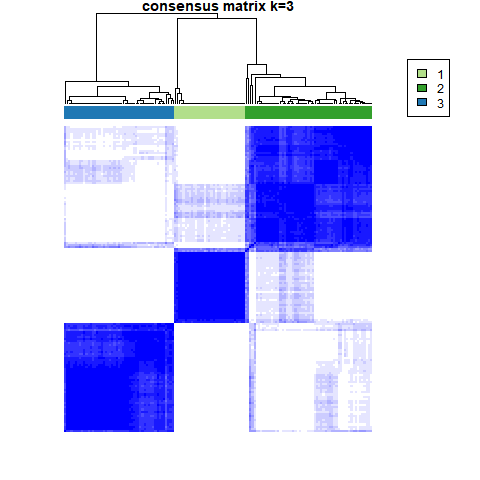

Supplement: Supplementary file 1 [file DataSheet_1.zip › raw data and Rcode for checking/Fig2/consensus003.png]

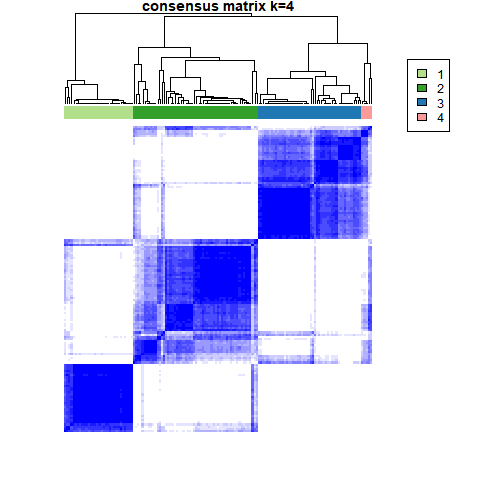

Supplement: Supplementary file 1 [file DataSheet_1.zip › raw data and Rcode for checking/Fig2/consensus004.png]

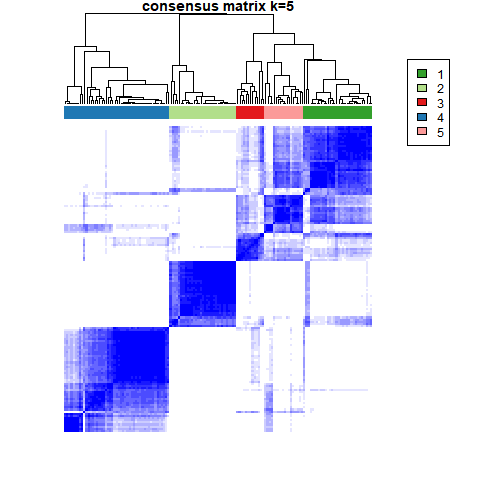

Supplement: Supplementary file 1 [file DataSheet_1.zip › raw data and Rcode for checking/Fig2/consensus005.png]

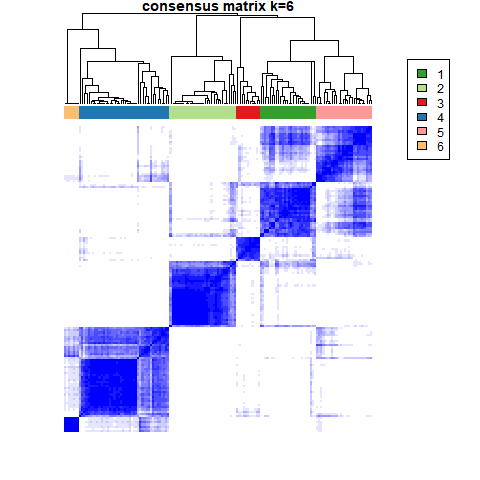

Supplement: Supplementary file 1 [file DataSheet_1.zip › raw data and Rcode for checking/Fig2/consensus006.png]

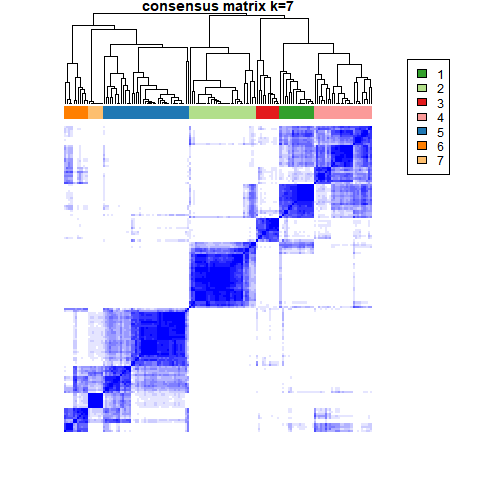

Supplement: Supplementary file 1 [file DataSheet_1.zip › raw data and Rcode for checking/Fig2/consensus007.png]

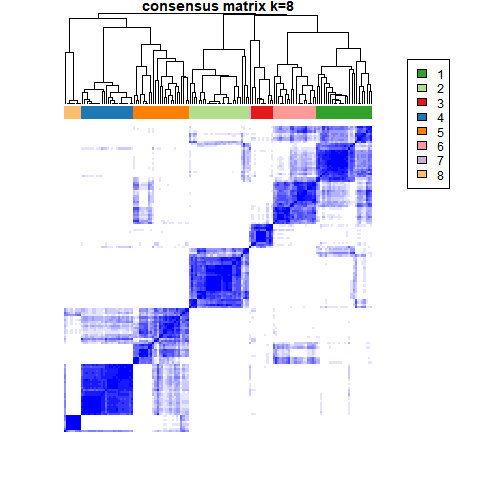

Supplement: Supplementary file 1 [file DataSheet_1.zip › raw data and Rcode for checking/Fig2/consensus008.png]

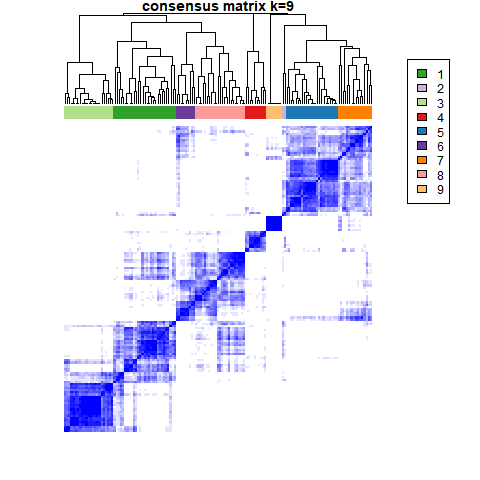

Supplement: Supplementary file 1 [file DataSheet_1.zip › raw data and Rcode for checking/Fig2/consensus009.png]

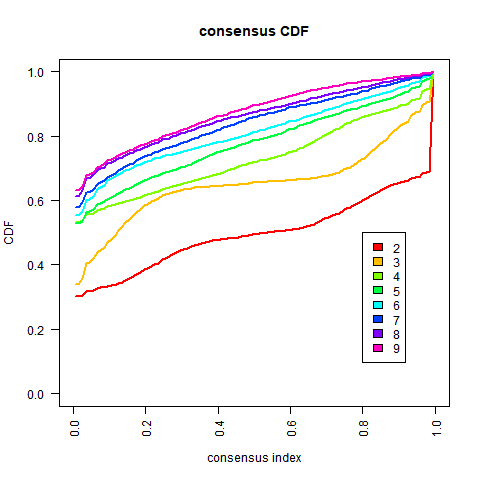

Supplement: Supplementary file 1 [file DataSheet_1.zip › raw data and Rcode for checking/Fig2/consensus010.png]

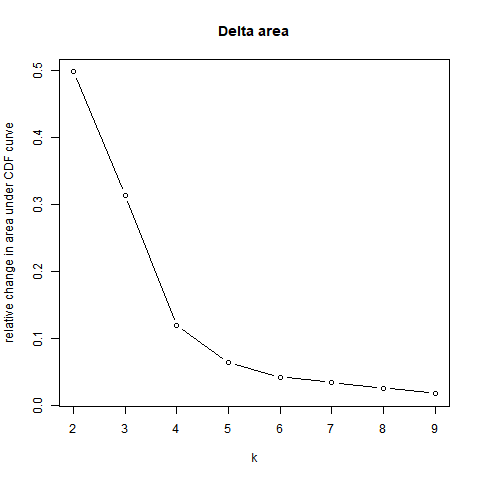

Supplement: Supplementary file 1 [file DataSheet_1.zip › raw data and Rcode for checking/Fig2/consensus011.png]

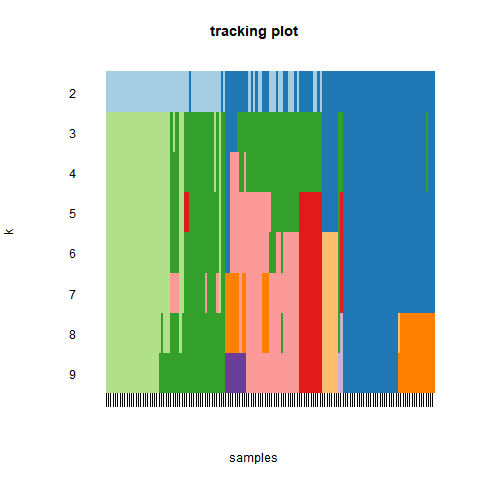

Supplement: Supplementary file 1 [file DataSheet_1.zip › raw data and Rcode for checking/Fig2/consensus012.png]

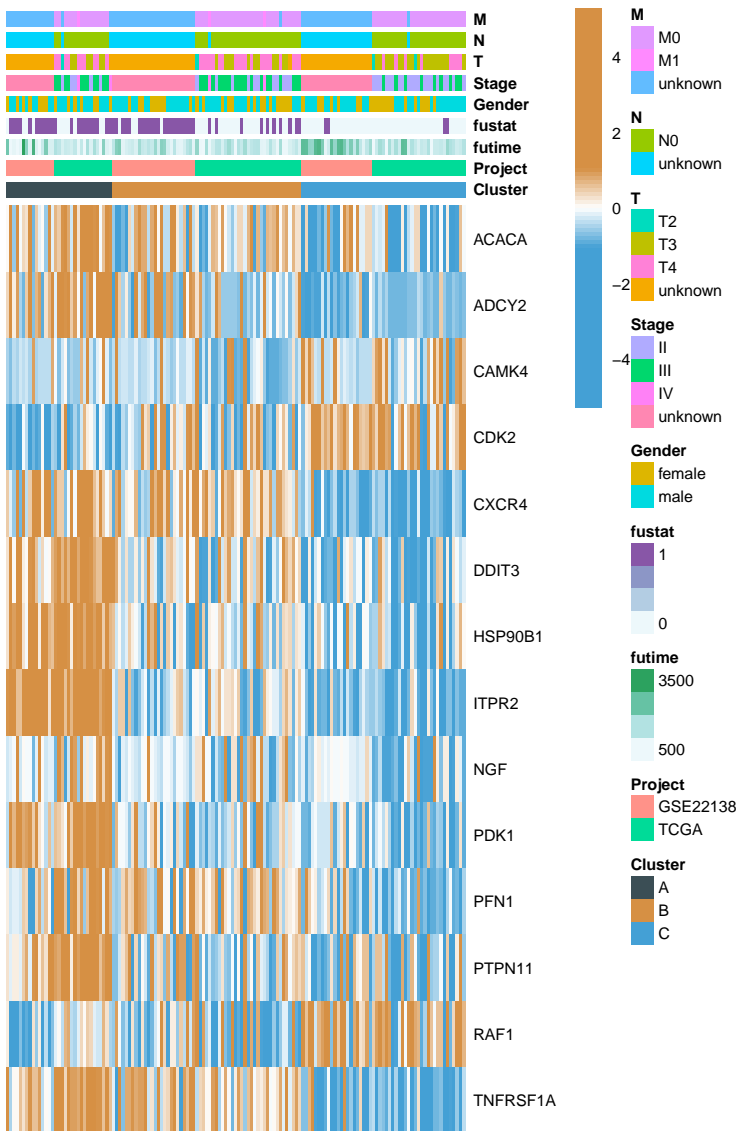

Supplement: Supplementary file 1 [file DataSheet_1.zip › raw data and Rcode for checking/Fig2/heatmap.pdf]

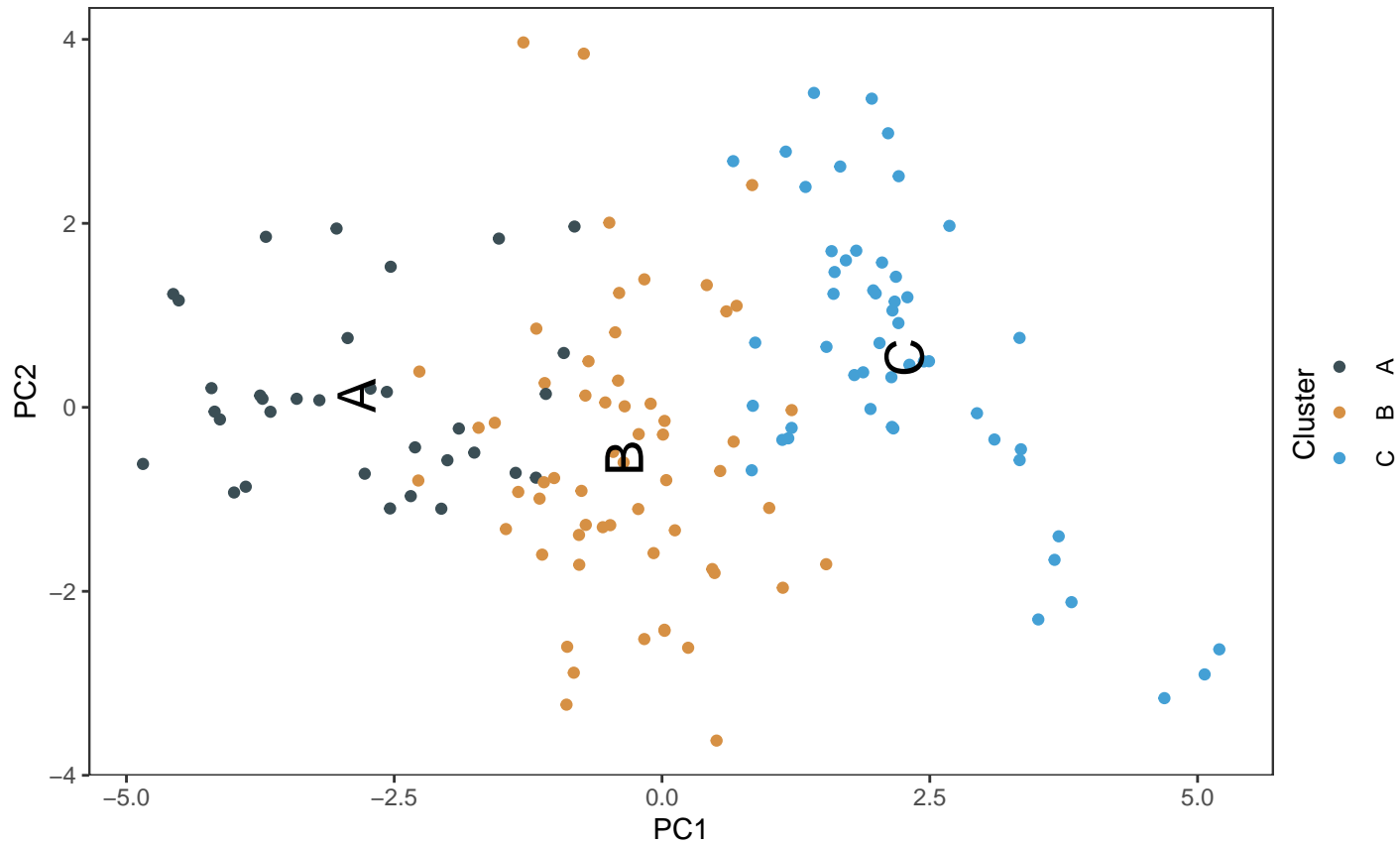

Supplement: Supplementary file 1 [file DataSheet_1.zip › raw data and Rcode for checking/Fig2/PCA.pdf]

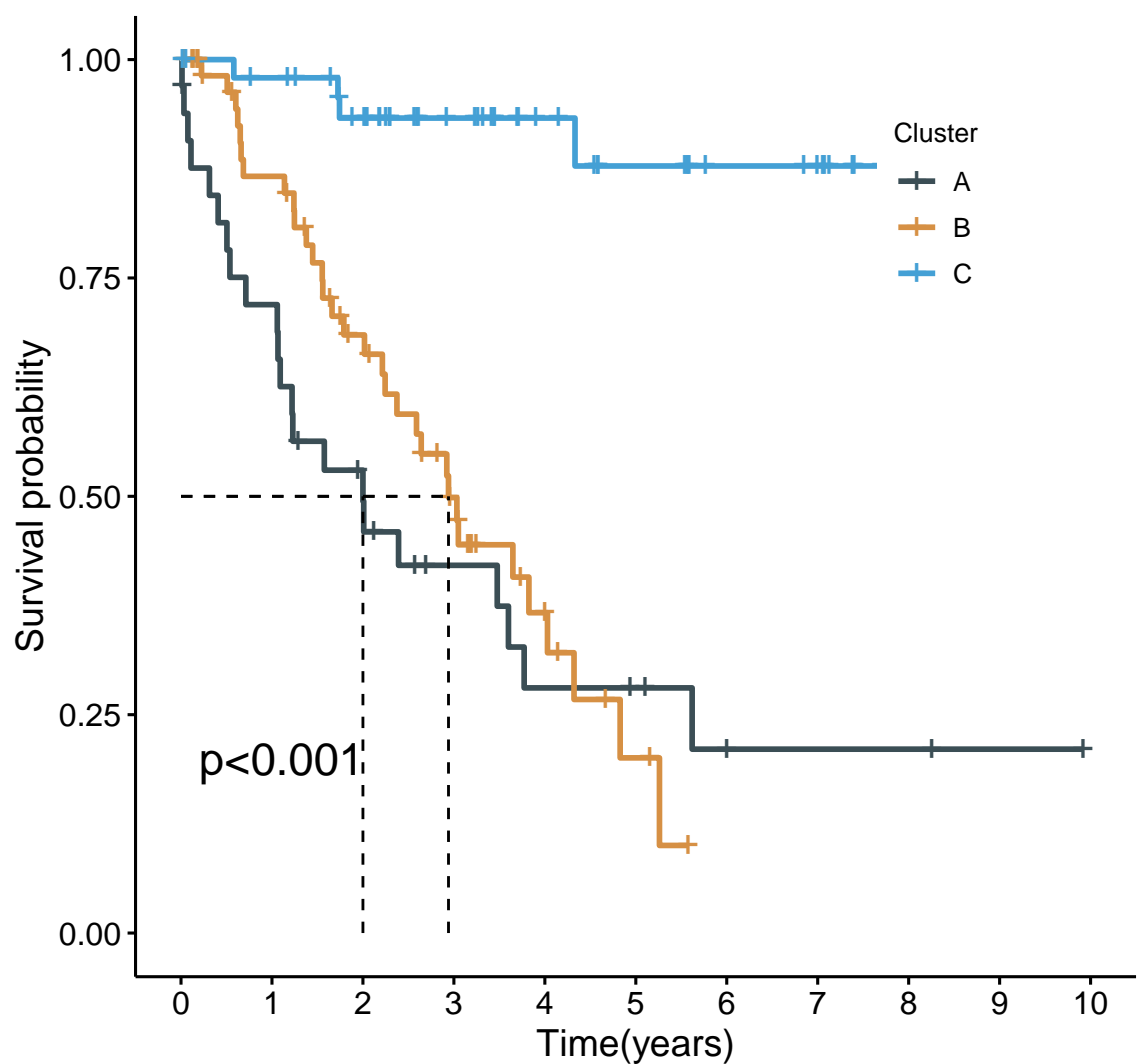

Number at risk

| Cluster | 0  | 1  | 2  | 3  | 4  | 5  | 6 | 7 | 8 | 9 | 10 |
|---------|----|----|----|----|----|----|---|---|---|---|----|
| A       | 33 | 23 | 15 | 9  | 6  | 5  | 3 | 2 | 2 | 1 | 0  |
| B       | 59 | 45 | 31 | 19 | 8  | 3  | 0 | 0 | 0 | 0 | 0  |
| C       | 51 | 46 | 39 | 27 | 18 | 13 | 9 | 7 | 0 | 0 | 0  |

Time(years)

Supplement: Supplementary file 1 [file DataSheet_1.zip › raw data and Rcode for checking/Fig2/survival.pdf]

Cluster    A    B    C

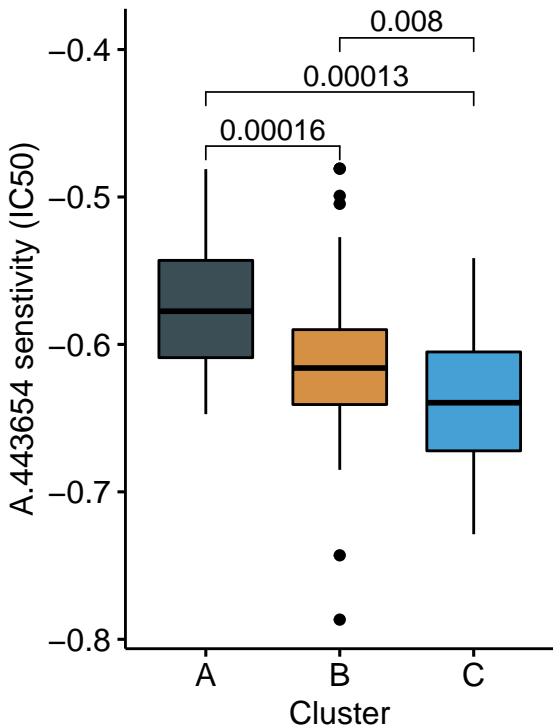

Supplement: Supplementary file 1 [file DataSheet_1.zip › raw data and Rcode for checking/Fig3/durgSenstivity.A.443654.pdf]

Cluster A B C

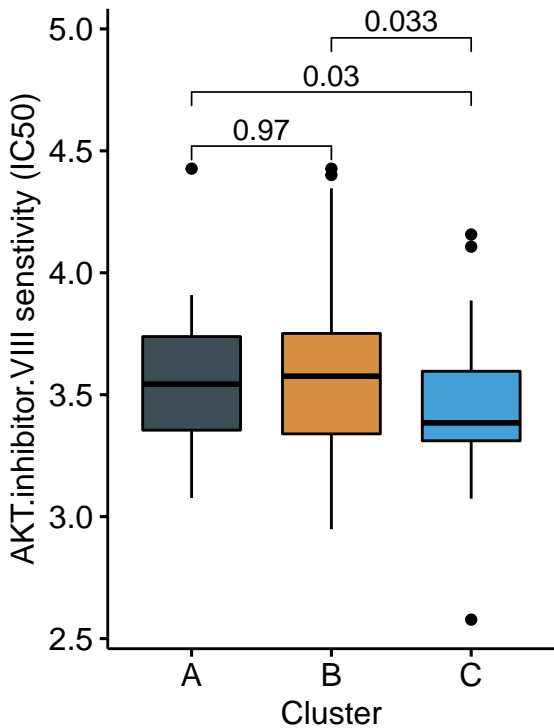

Supplement: Supplementary file 1 [file DataSheet_1.zip › raw data and Rcode for checking/Fig3/durgSenstivity.AKT.inhibitor.VIII.pdf]

Cluster A B C

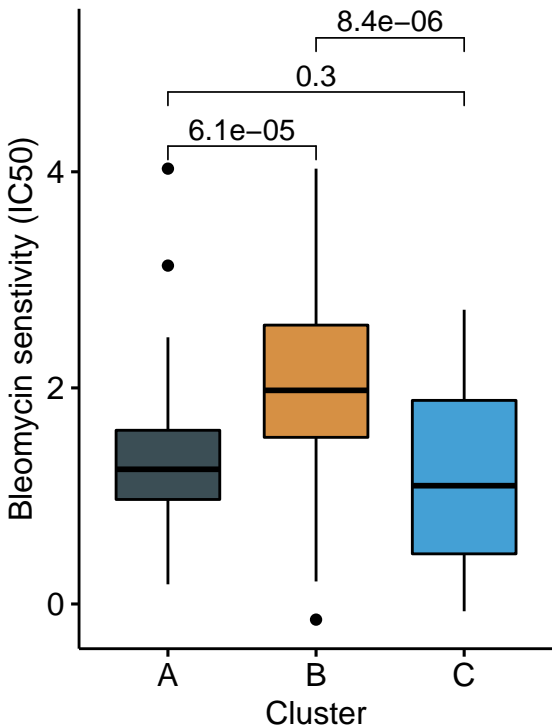

Supplement: Supplementary file 1 [file DataSheet_1.zip › raw data and Rcode for checking/Fig3/durgSenstivity.Bleomycin.pdf]

Cluster 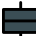 A 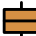 B 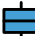 C

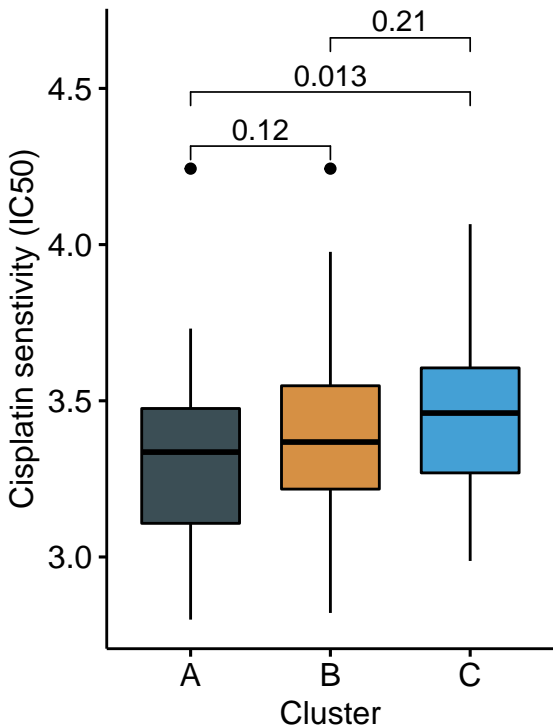

Supplement: Supplementary file 1 [file DataSheet_1.zip › raw data and Rcode for checking/Fig3/durgSenstivity.Cisplatin.pdf]

Cluster 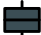 A 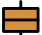 B 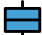 C

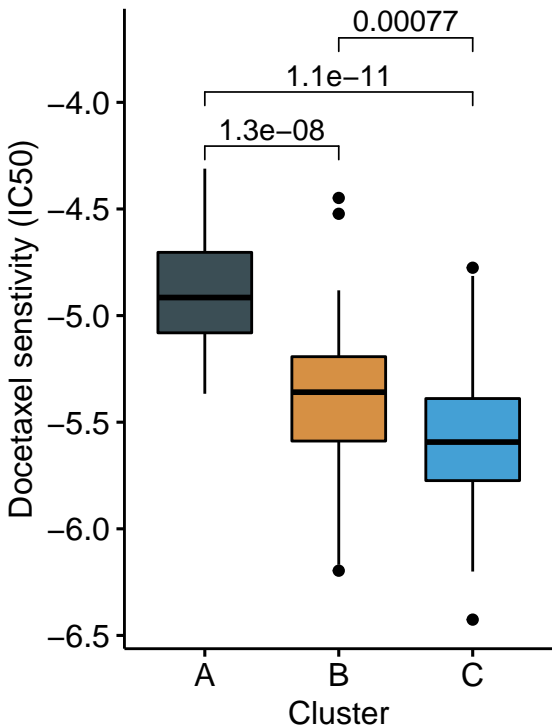

Supplement: Supplementary file 1 [file DataSheet_1.zip › raw data and Rcode for checking/Fig3/durgSenstivity.Docetaxel.pdf]

Cluster    A    B    C

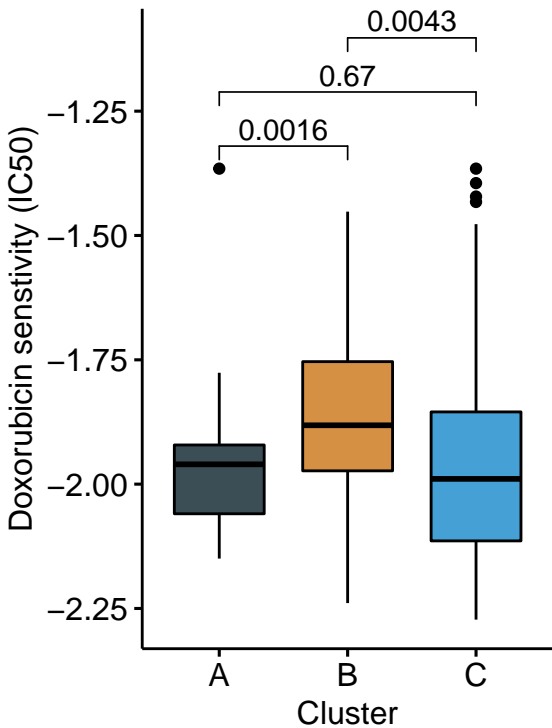

Supplement: Supplementary file 1 [file DataSheet_1.zip › raw data and Rcode for checking/Fig3/durgSenstivity.Doxorubicin.pdf]

Cluster A B C

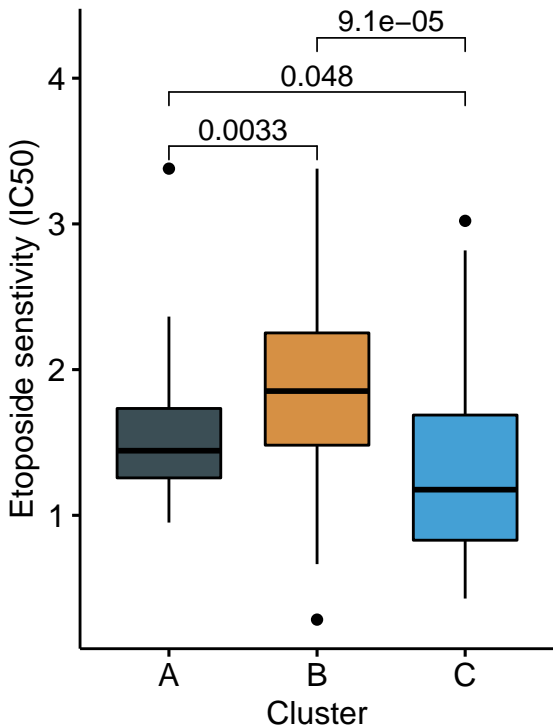

Supplement: Supplementary file 1 [file DataSheet_1.zip › raw data and Rcode for checking/Fig3/durgSenstivity.Etoposide.pdf]

Cluster 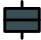 A 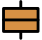 B 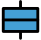 C

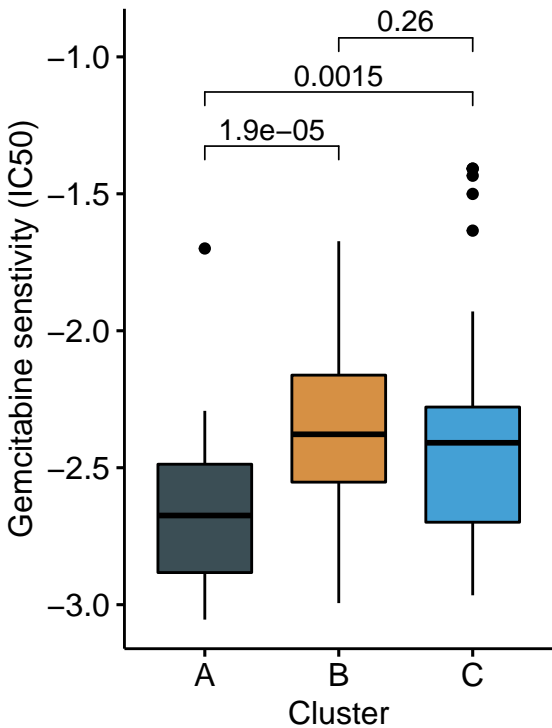

Supplement: Supplementary file 1 [file DataSheet_1.zip › raw data and Rcode for checking/Fig3/durgSenstivity.Gemcitabine.pdf]

Cluster    A    B    C

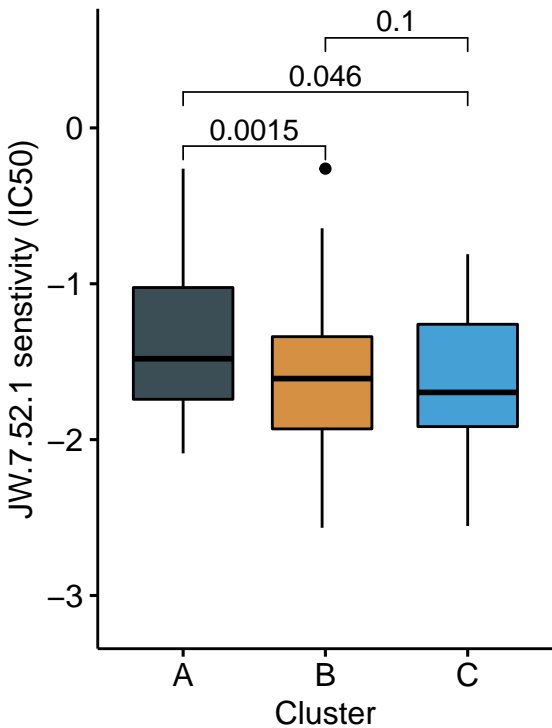

Supplement: Supplementary file 1 [file DataSheet_1.zip › raw data and Rcode for checking/Fig3/durgSenstivity.JW.7.52.1.pdf]

Cluster    A    B    C

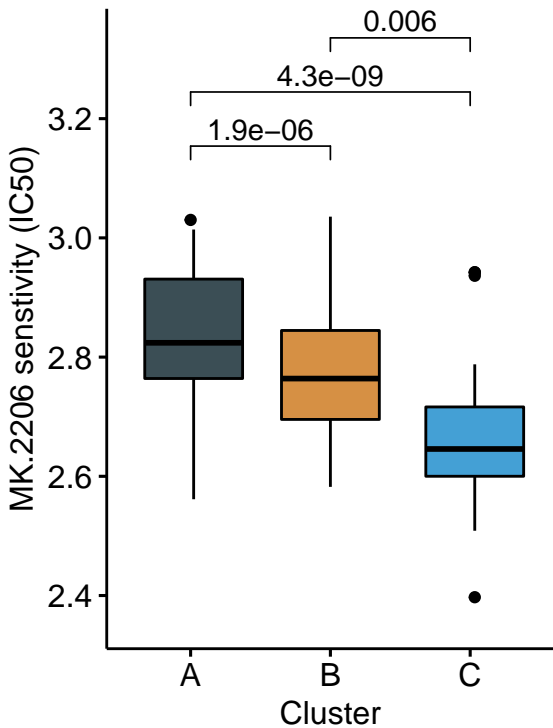

Supplement: Supplementary file 1 [file DataSheet_1.zip › raw data and Rcode for checking/Fig3/durgSenstivity.MK.2206.pdf]

Cluster A B C

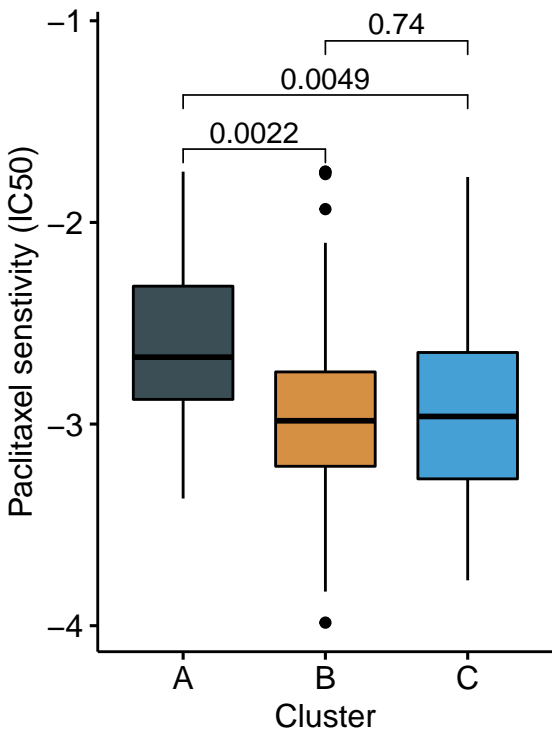

Supplement: Supplementary file 1 [file DataSheet_1.zip › raw data and Rcode for checking/Fig3/durgSenstivity.Paclitaxel.pdf]

Cluster C B A

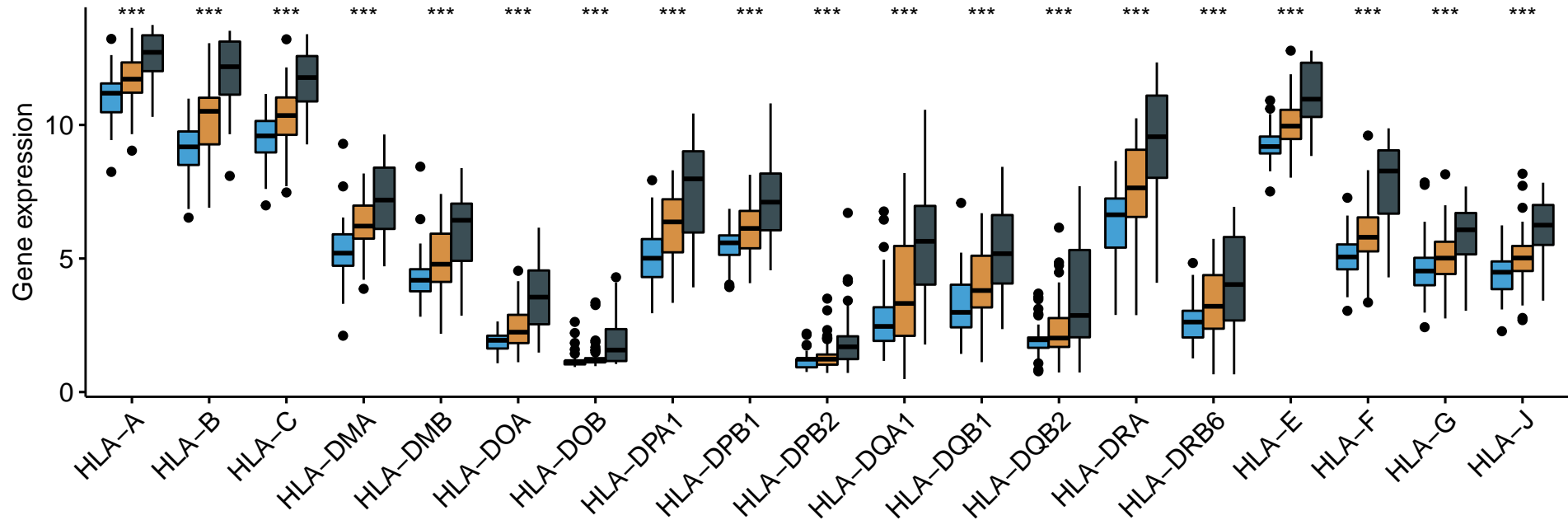

Supplement: Supplementary file 1 [file DataSheet_1.zip › raw data and Rcode for checking/Fig3/HLA.pdf]

Cluster C B A

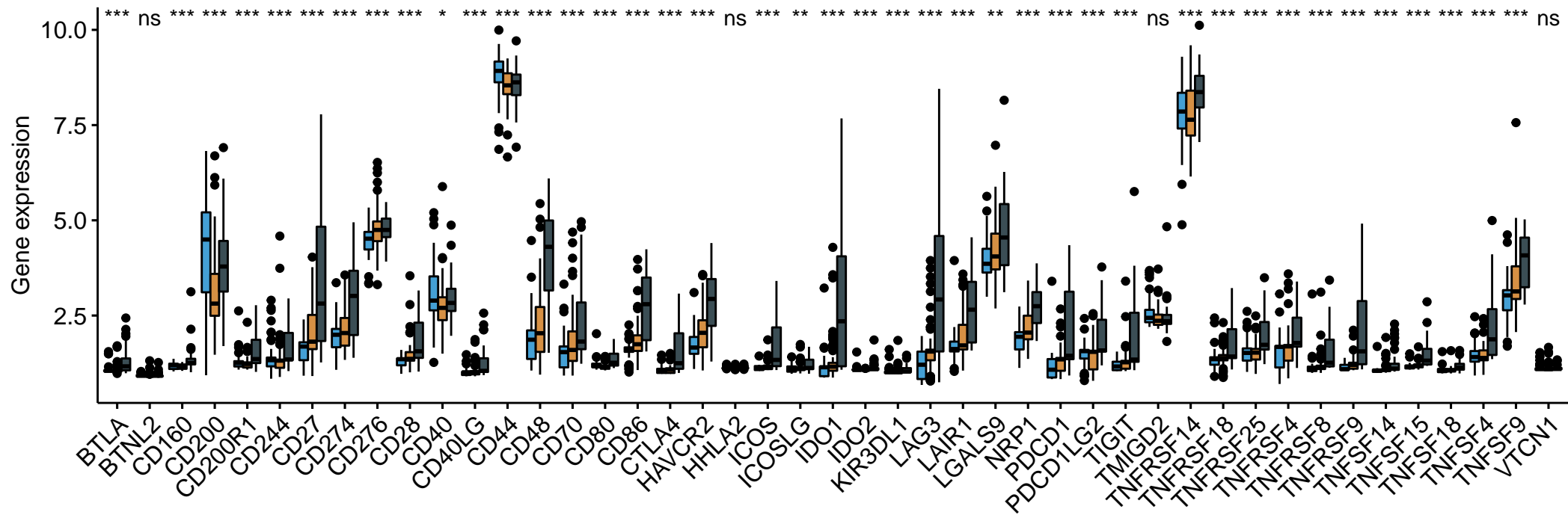

Supplement: Supplementary file 1 [file DataSheet_1.zip › raw data and Rcode for checking/Fig3/ICI.pdf]

Immune infiltration

Cluster A B C

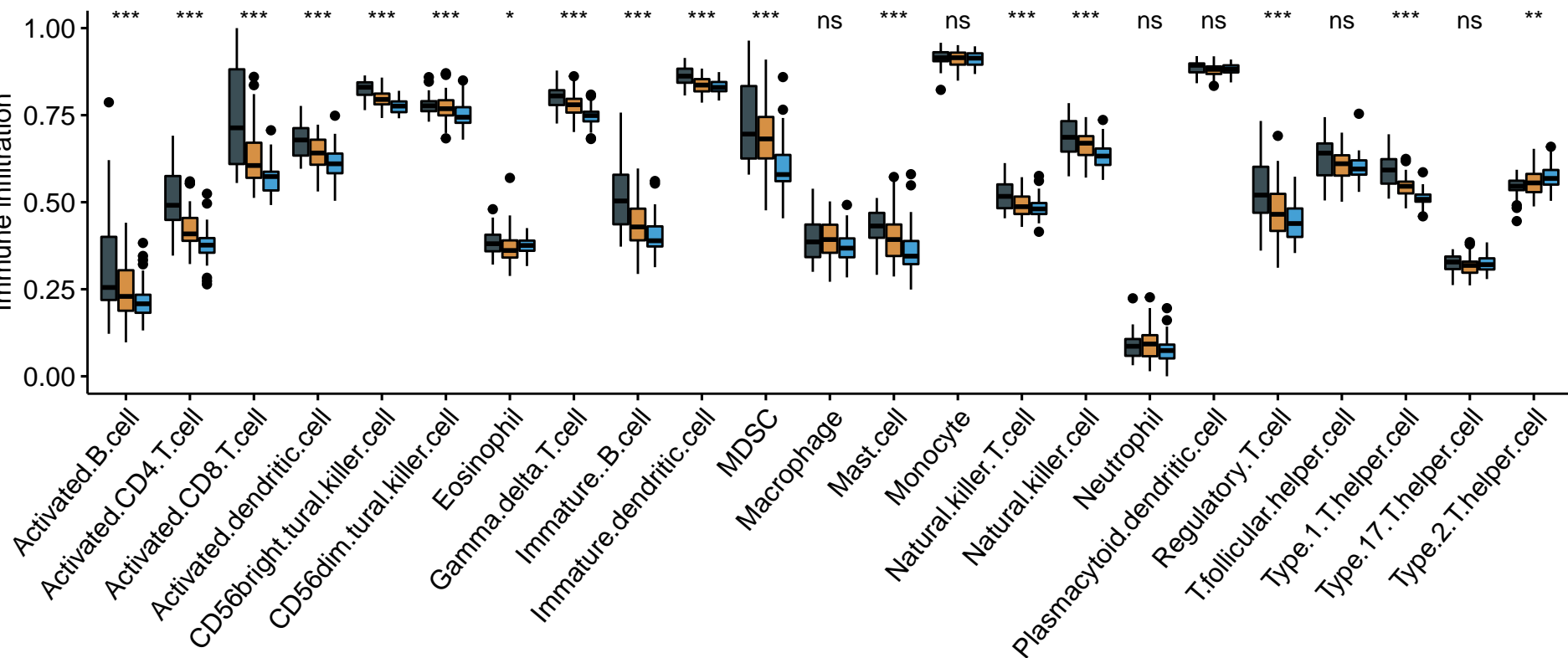

Supplement: Supplementary file 1 [file DataSheet_1.zip › raw data and Rcode for checking/Fig3/ssGSEA.pdf]

Cluster C B A

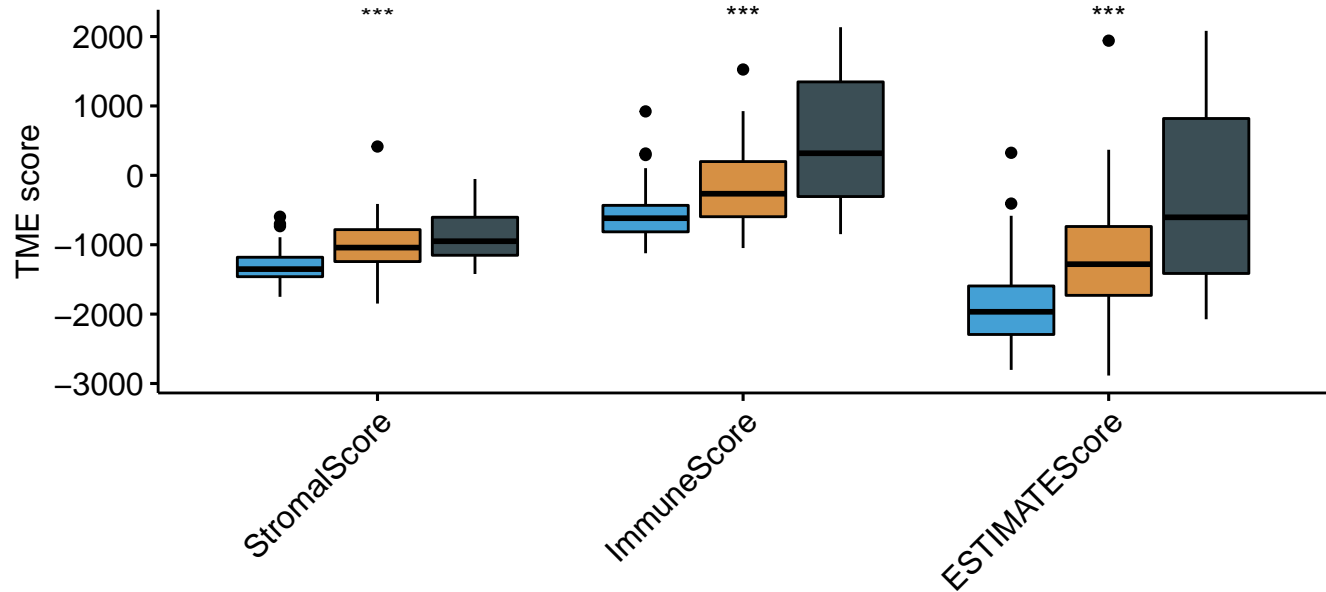

Supplement: Supplementary file 1 [file DataSheet_1.zip › raw data and Rcode for checking/Fig3/TME.pdf]

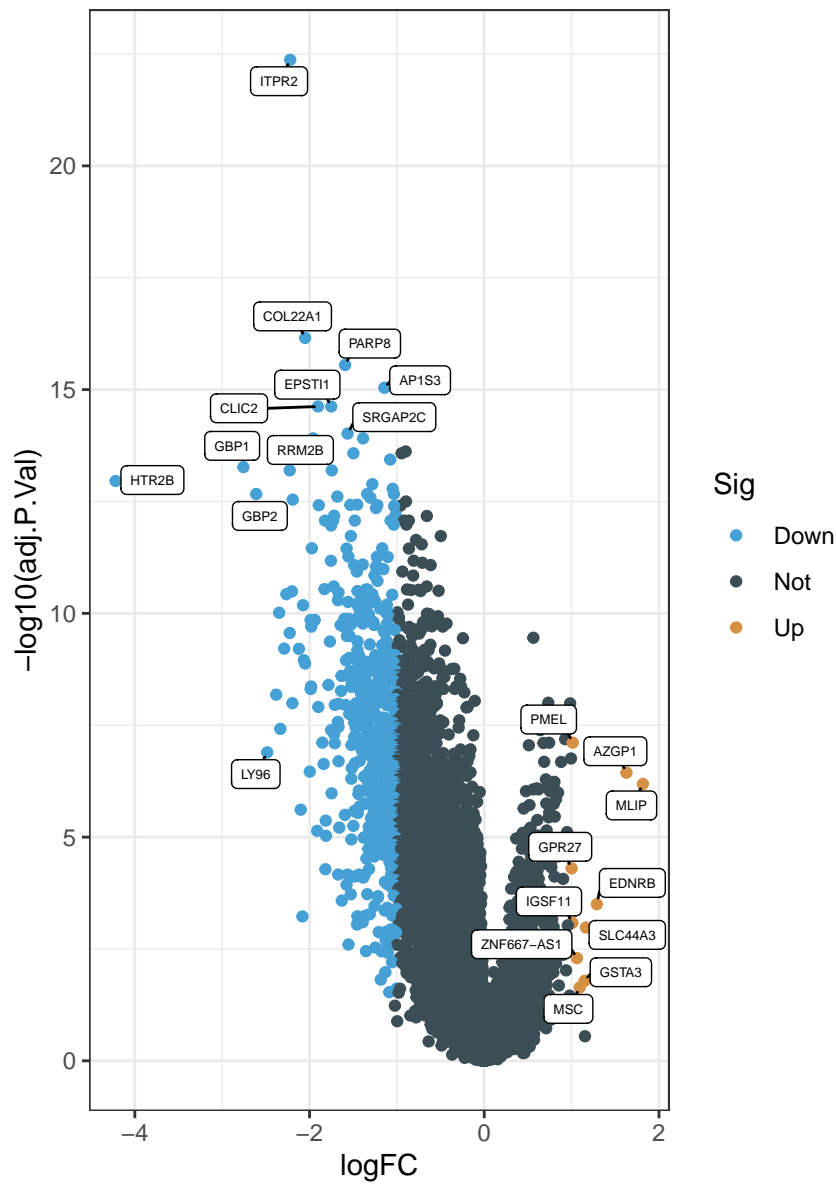

Supplement: Supplementary file 1 [file DataSheet_1.zip › raw data and Rcode for checking/Fig4/vol-B-A.pdf]

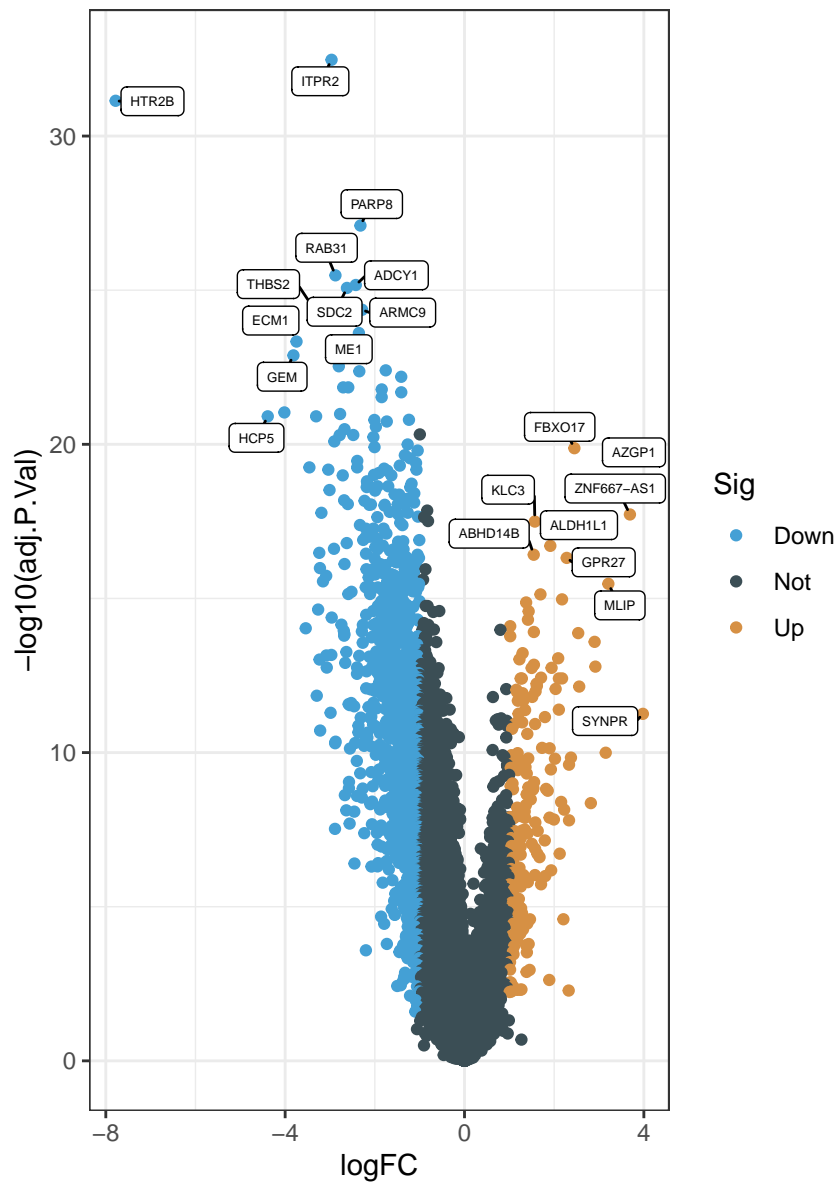

Supplement: Supplementary file 1 [file DataSheet_1.zip › raw data and Rcode for checking/Fig4/vol-C-A.pdf]

$-\log_{10}(\text{adj.P.Val})$

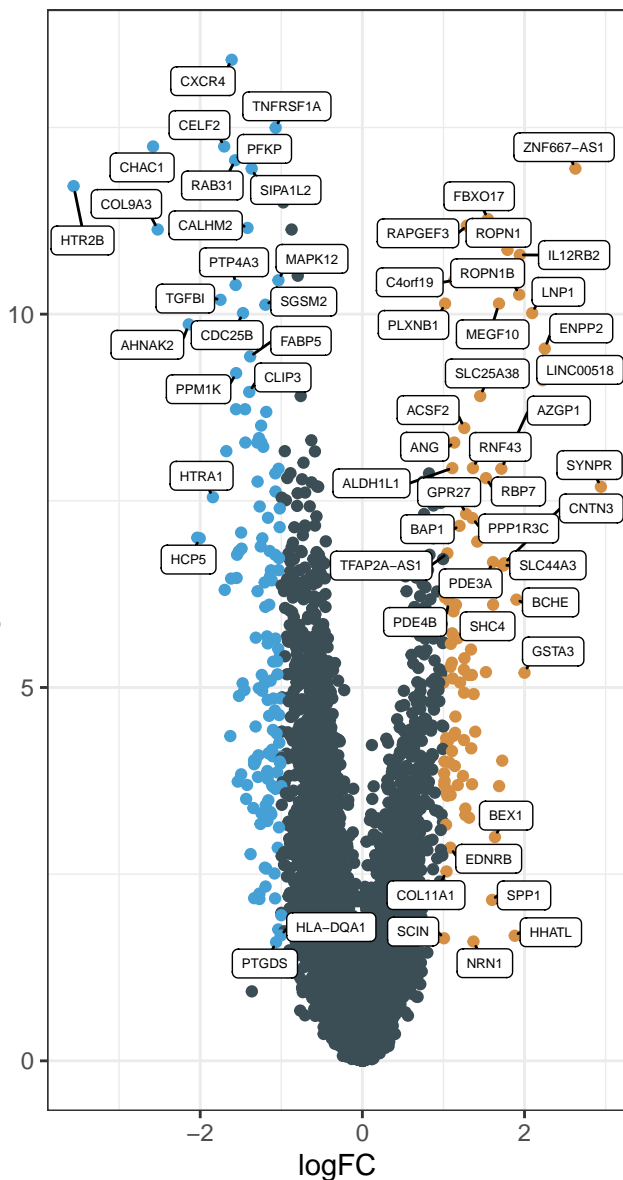

Supplement: Supplementary file 1 [file DataSheet_1.zip › raw data and Rcode for checking/Fig4/vol-C-B.pdf]

# Altered in 24 (92.31%) of 26 samples.

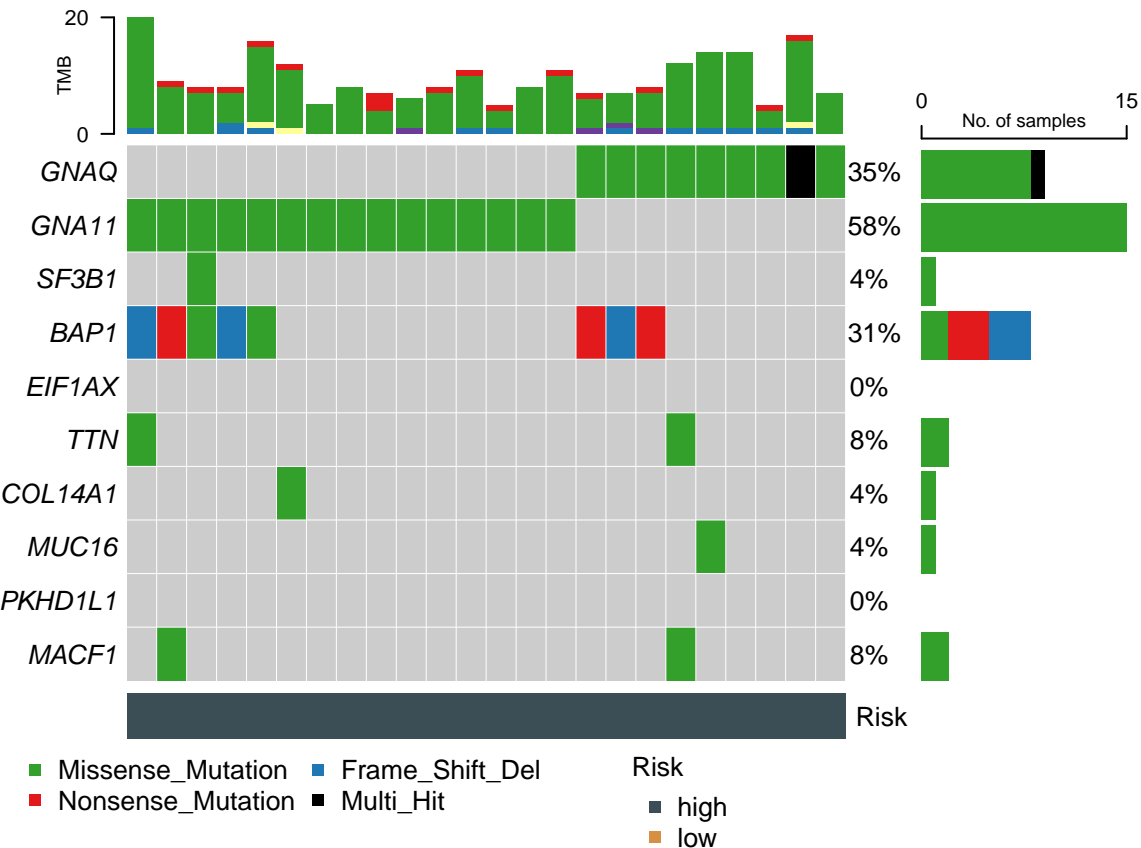

Supplement: Supplementary file 1 [file DataSheet_1.zip › raw data and Rcode for checking/Fig6/high.pdf]

# Altered in 53 (98.15%) of 54 samples.

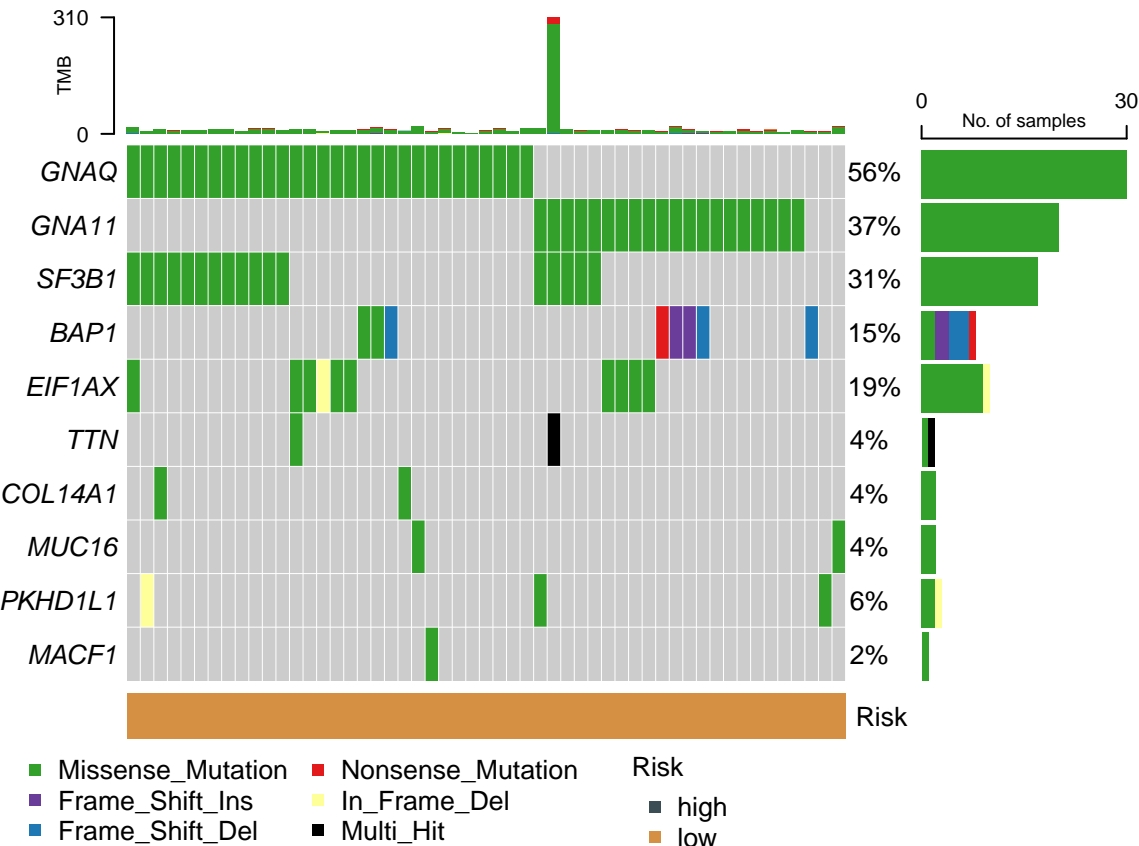

Supplement: Supplementary file 1 [file DataSheet_1.zip › raw data and Rcode for checking/Fig6/low.pdf]
