# Supplementary material for: PI3K/AKT/mTOR pathway-derived risk score exhibits correlation with immune infiltration in uveal melanoma patients
Source: Front Oncol. 2023 Apr 20;13:1167930. doi: 10.3389/fonc.2023.1167930 (PMC10157141; doi:10.3389/fonc.2023.1167930)
Supplement: Supplementary file 3 [file Table_2.docx]

| **Supplementary files 2: The performance of 76 predictive models in training, testing and the whole cohorts.** | | | |
| --- | --- | --- | --- |
| **Model** | **TCGA-UVM** | **GSE22138** | **All datasets** |
| Lasso+StepCox[both] | 0.83234714 | 0.685147159 | 0.733386076 |
| survivalSVM | 0.724852071 | 0.691307324 | 0.695015823 |
| Ridge | 0.794871795 | 0.737166324 | 0.758900316 |
| Lasso+survivalSVM | 0.784023669 | 0.703627652 | 0.730814873 |
| SuperPC | 0.744575937 | 0.68788501 | 0.70846519 |
| Enet[alpha=0.1] | 0.812623274 | 0.729637235 | 0.761669304 |
| Enet[alpha=0.2] | 0.821499014 | 0.726214921 | 0.761669304 |
| Enet[alpha=0.3] | 0.821499014 | 0.72347707 | 0.76068038 |
| Enet[alpha=0.4] | 0.827416174 | 0.724161533 | 0.762262658 |
| Enet[alpha=0.5] | 0.82938856 | 0.72073922 | 0.760087025 |
| Enet[alpha=0.6] | 0.837278107 | 0.710472279 | 0.755537975 |
| Enet[alpha=0.8] | 0.836291913 | 0.704996578 | 0.752768987 |
| Enet[alpha=0.9] | 0.813609467 | 0.709103354 | 0.746044304 |
| Lasso | 0.814595661 | 0.70568104 | 0.743868671 |
| Enet[alpha=0.7] | 0.840236686 | 0.707049966 | 0.754153481 |
| Lasso+plsRcox | 0.788954635 | 0.691307324 | 0.73397943 |
| Lasso+StepCox[forward] | 0.848126233 | 0.694729637 | 0.74505538 |
| RSF+survivalSVM | 0.738658777 | 0.672142368 | 0.696993671 |
| StepCox[forward] | 0.867850099 | 0.64476386 | 0.725870253 |
| plsRcox | 0.801775148 | 0.688569473 | 0.735363924 |
| RSF+Ridge | 0.769230769 | 0.702258727 | 0.726265823 |
| RSF+Enet[alpha=0.1] | 0.775147929 | 0.704312115 | 0.728045886 |
| Lasso+SuperPC | 0.784023669 | 0.707734428 | 0.746439873 |
| RSF+plsRcox | 0.747534517 | 0.692676249 | 0.710047468 |
| RSF+StepCox[forward] | 0.800788955 | 0.65982204 | 0.717563291 |
| RSF+Enet[alpha=0.2] | 0.785009862 | 0.700205339 | 0.729628165 |
| RSF+Enet[alpha=0.3] | 0.787968442 | 0.700205339 | 0.729825949 |
| RSF+Enet[alpha=0.6] | 0.791913215 | 0.696098563 | 0.730221519 |
| RSF+Lasso | 0.797830375 | 0.691991786 | 0.727254747 |
| RSF+Enet[alpha=0.7] | 0.791913215 | 0.694729637 | 0.728441456 |
| RSF+Enet[alpha=0.5] | 0.787968442 | 0.696098563 | 0.728441456 |
| RSF+Enet[alpha=0.9] | 0.794871795 | 0.691991786 | 0.727254747 |
| RSF+Enet[alpha=0.4] | 0.789940828 | 0.696098563 | 0.730221519 |
| RSF+Enet[alpha=0.8] | 0.789940828 | 0.691991786 | 0.727056962 |
| RSF+StepCox[both] | 0.793885602 | 0.684462697 | 0.721123418 |
| RSF+StepCox[backward] | 0.793885602 | 0.684462697 | 0.721123418 |
| StepCox[both]+Ridge | 0.814595661 | 0.667351129 | 0.732594937 |
| StepCox[backward]+Ridge | 0.814595661 | 0.667351129 | 0.732594937 |
| StepCox[both]+plsRcox | 0.689349112 | 0.65982204 | 0.66119462 |
| StepCox[backward]+plsRcox | 0.689349112 | 0.65982204 | 0.66119462 |
| StepCox[both]+Enet[alpha=0.9] | 0.816568047 | 0.668035592 | 0.732792722 |
| StepCox[backward]+Enet[alpha=0.9] | 0.815581854 | 0.668035592 | 0.732397152 |
| StepCox[both]+Enet[alpha=0.1] | 0.814595661 | 0.667351129 | 0.732397152 |
| StepCox[backward]+Enet[alpha=0.1] | 0.814595661 | 0.667351129 | 0.732397152 |
| StepCox[both]+Enet[alpha=0.8] | 0.815581854 | 0.668035592 | 0.732397152 |
| StepCox[backward]+Enet[alpha=0.8] | 0.815581854 | 0.668035592 | 0.732397152 |
| StepCox[both]+Enet[alpha=0.2] | 0.815581854 | 0.667351129 | 0.732594937 |
| StepCox[backward]+Enet[alpha=0.2] | 0.815581854 | 0.667351129 | 0.732594937 |
| StepCox[both]+Lasso | 0.816568047 | 0.668035592 | 0.732594937 |
| StepCox[backward]+Lasso | 0.816568047 | 0.668035592 | 0.732594937 |
| StepCox[both]+Enet[alpha=0.6] | 0.816568047 | 0.668035592 | 0.732792722 |
| StepCox[backward]+Enet[alpha=0.6] | 0.816568047 | 0.668035592 | 0.732792722 |
| StepCox[both]+Enet[alpha=0.7] | 0.815581854 | 0.668035592 | 0.732792722 |
| StepCox[backward]+Enet[alpha=0.7] | 0.816568047 | 0.668035592 | 0.732792722 |
| Lasso+StepCox[backward] | 0.83234714 | 0.685147159 | 0.733386076 |
| StepCox[both] | 0.815581854 | 0.667351129 | 0.732594937 |
| StepCox[backward] | 0.815581854 | 0.667351129 | 0.732594937 |
| StepCox[both]+Enet[alpha=0.4] | 0.815581854 | 0.667351129 | 0.732594937 |
| StepCox[backward]+Enet[alpha=0.4] | 0.816568047 | 0.668035592 | 0.732990506 |
| StepCox[both]+Enet[alpha=0.3] | 0.815581854 | 0.667351129 | 0.732594937 |
| StepCox[backward]+Enet[alpha=0.3] | 0.815581854 | 0.667351129 | 0.732594937 |
| StepCox[both]+Enet[alpha=0.5] | 0.816568047 | 0.668035592 | 0.732990506 |
| StepCox[backward]+Enet[alpha=0.5] | 0.816568047 | 0.668035592 | 0.732792722 |
| RSF+SuperPC | 0.744575937 | 0.68788501 | 0.70846519 |
| RSF | 0.929980276 | 0.694729637 | 0.797666139 |
| Lasso+GBM | 0.862919132 | 0.67761807 | 0.763647152 |
| RSF+GBM | 0.834319527 | 0.666666667 | 0.734177215 |
| GBM | 0.912228797 | 0.696783025 | 0.79647943 |
| StepCox[both]+survivalSVM | 0.695266272 | 0.661875428 | 0.672468354 |
| StepCox[backward]+survivalSVM | 0.695266272 | 0.661875428 | 0.672468354 |
| Lasso+RSF | 0.929980276 | 0.679671458 | 0.789754747 |
| StepCox[both]+GBM | 0.908284024 | 0.691991786 | 0.786787975 |
| StepCox[backward]+GBM | 0.861932939 | 0.699863107 | 0.769284019 |
| StepCox[both]+RSF | 0.911242604 | 0.702258727 | 0.789359177 |
| StepCox[backward]+RSF | 0.91321499 | 0.702600958 | 0.789655854 |
